# Multifunctional carbonized nanogels to treat lethal acute hepatopancreatic necrosis disease

**DOI:** 10.1186/s12951-021-01194-8

**Published:** 2021-12-24

**Authors:** Shao-Chieh Yen, Ju-Yi Mao, Hung-Yun Lin, Huai-Ting Huang, Scott G. Harroun, Amit Nain, Huan-Tsung Chang, Han-You Lin, Li-Li Chen, Chih-Ching Huang, Han-Jia Lin

**Affiliations:** 1 Department of Bioscience and Biotechnology, National Taiwan Ocean University, Keelung, 202301 Taiwan; 2 Doctoral Degree Program in Marine Biotechnology, National Taiwan Ocean University, Keelung, 202301 Taiwan; 3 Department of Aquaculture, National Taiwan Ocean University, Keelung, 202301 Taiwan; 4 Department of Chemistry, Université de Montréal, Montreal, QC H3C 3J7 Canada; 5 Department of Chemistry, National Taiwan University, Taipei, 10617 Taiwan; 6 Department of Veterinary Medicine, School of Veterinary Medicine, National Taiwan University, Taipei, 10617 Taiwan; 7 Institute of Marine Biology, National Taiwan Ocean University, Keelung, 202301 Taiwan; 8 Center of Excellence for the Oceans, National Taiwan Ocean University, Keelung, 202301 Taiwan; 9 School of Pharmacy, College of Pharmacy, Kaohsiung Medical University, Kaohsiung, 80708 Taiwan

**Keywords:** Carbon nanogels, Antimicrobial agents, Antibiotic overuse, *Vibrio*, Toxin neutralization

## Abstract

**Background:**

Shrimp aquaculture has suffered huge economic losses over the past decade due to the outbreak of acute hepatopancreatic necrosis disease (AHPND), which is mainly caused by the bacteria *Vibrio parahaemolyticus* (*V. parahaemolyticus*) with the virulence pVA1 plasmid, which encodes a secretory photorhabdus insect-related (Pir) toxin composed of PirA and PirB proteins. The Pir toxin mainly attacks the hepatopancreas, a major metabolic organ in shrimp, thereby causing necrosis and loss of function. The pandemic of antibiotic-resistant strains makes the impact worse.

**Methods:**

Mild pyrolysis of a mixture of polysaccharide dextran 70 and the crosslinker 1,8-diaminooctane at 180 ℃ for 3 h to form carbonized nanogels (DAO/DEX-CNGs) through controlled cross-linking and carbonization. The multifunctional therapeutic CNGs inherit nanogel-like structures and functional groups from their precursor molecules.

**Results:**

DAO/DEX-CNGs manifest broad-spectrum antibacterial activity against *Vibrio parahaemolyticus* responsible for AHPND and even multiple drug-resistant strains. The polymer-like structures and functional groups on graphitic-carbon within the CNGs exhibit multiple treatment effects, including disruption of bacterial membranes, elevating bacterial oxidative stress, and neutralization of PirAB toxins. The inhibition of *Vibrio* in the midgut of infected shrimp, protection of hepatopancreas tissue from Pir toxin, and suppressing overstimulation of the immune system in severe *V. parahaemolyticus* infection, revealing that CNGs can effectively guard shrimp from *Vibrio* invasion. Moreover, shrimps fed with DAO/DEX-CNGs were carefully examined, such as the expression of the immune-related genes, hepatopancreas biopsy, and intestinal microbiota. Few adverse effects on shrimps were observed.

**Conclusion:**

Our work proposes brand-new applications of multifunctional carbon-based nanomaterials as efficient anti-*Vibrio* agents in the aquatic industry that hold great potential as feed additives to reduce antibiotic overuse in aquaculture.

**Graphical Abstract:**

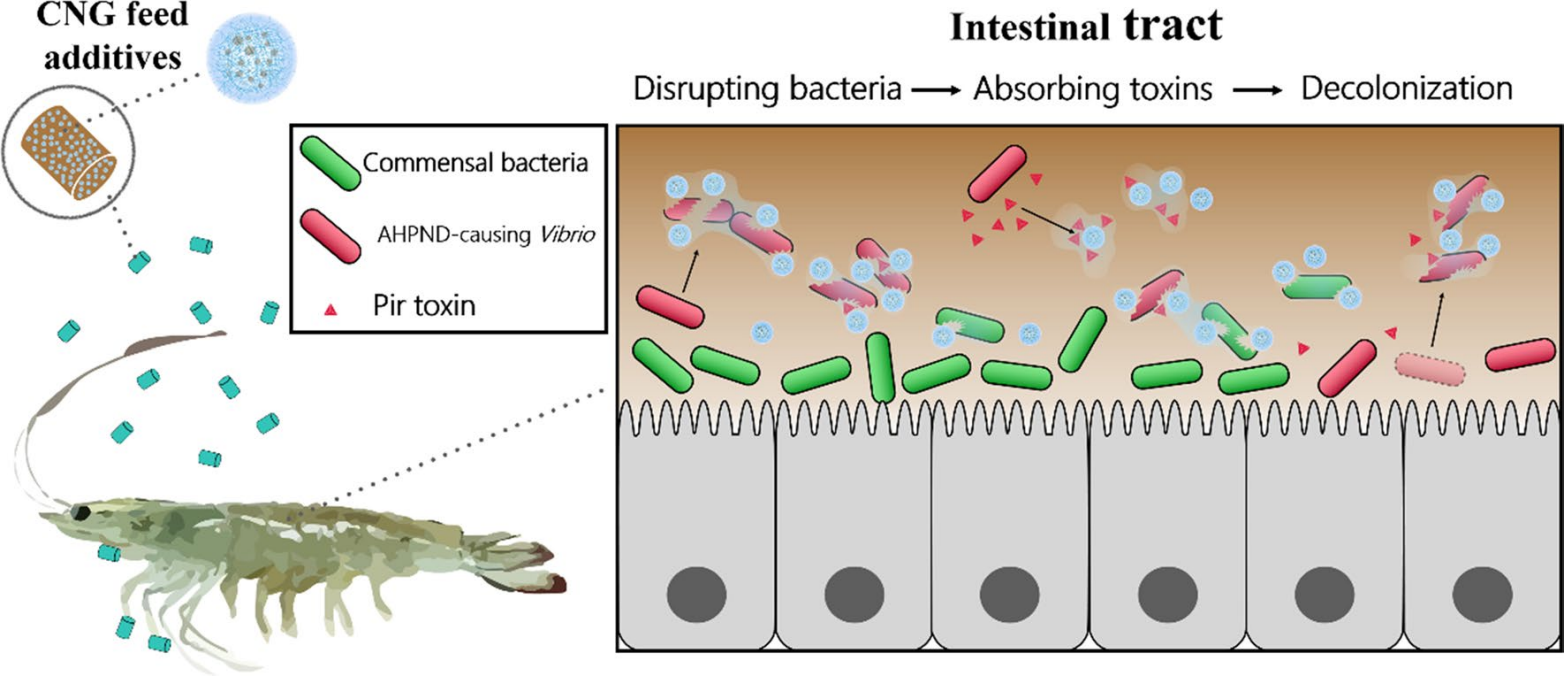

**Supplementary Information:**

The online version contains supplementary material available at 10.1186/s12951-021-01194-8.

## Background

Antibiotic abuse in healthcare and farming is a serious issue that threatens ecosystems and human health [1]. With the increasing demand for animal-based products, modern intensive farming relies on antibiotics to prevent infectious disease outbreaks [2]. External factors, such as globalization and climate change, have increased the requirement of animal antibiotics [3]. An analysis by the United States Food and Drug Administration has shown that up to 80% of antibiotics prescribed worldwide are administered to farm animals [4]. However, most are not authorized by medical professionals and are delivered indiscriminately for growth promotion [5]. This abuse of antibiotics can engender multidrug-resistant bacteria, which are of grave concern for the livestock industry and human health (e.g., transmission of resistant zoonotic pathogens) [6].

The total global production of the shrimp and prawn farming industries exceeded 4.15 million tons (worth > US$28 billion) in 2018 [7]. Among them, whiteleg shrimp (*Litopenaeus vannamei*) farming accounts for approximately US$20 billion of the global market. However, owing to fatal outbreaks of infectious diseases, such as white spot disease, hepatopancreatic microsporidiosis, and acute hepatopancreatic necrosis disease (AHPND), the aquaculture industry has suffered huge economic losses in recent years [8]. AHPND was first reported in China and Vietnam in 2010, and extended to Mexico just 3 years later. Since then, AHPND has spread to shrimp aquaculture ponds worldwide [9, 10]. According to statistics from the Food and Agriculture Organization of United Nations (FAO), between 2010 and 2016, AHPND was responsible for a colossal loss of US$44 billion in the global shrimp farming industry [9]. To date, AHPND-caused economic losses have been estimated as USD 7 billion annually [11, 12].

In 2013, it was discovered that the cause of AHPND is *Vibrio parahaemolyticus*, a zoonotic seafood-borne bacterial pathogen [13]. Although no cases of AHPND affecting humans have occurred yet, other *Vibrio* species can cause gastroenteritis and even sepsis in humans and aquatic animals [14]. Moreover, the AHPND-causing *V. parahaemolyticus* has inherited several virulence factors, including plasmid pVA1, which encodes for a secretory *Photorhabdus* insect-related (Pir) toxin composed of PirA and PirB proteins, through horizontal gene transfer (HGT). The Pir toxin mainly attacks the hepatopancreas, thereby causing necrosis and functional loss in this major metabolic organ of shrimp [15]. Even in cases where antibiotics eliminate the pathogenic bacteria, the toxins remain fatal [16]. Additionally, *V. parahaemolyticus* can acquire antibiotic resistance genes through HGT [17]. Therefore, AHPND treatment has become unpredictable and exasperating, and even the FAO admits that there is no “silver bullet” against AHPND [18].

Owing to the increased regulation of antibiotic use in farm animals and concerns regarding drug-resistant bacteria, novel nanomaterial-based antimicrobials have been developed for the effective control of pathogens [19]. For example, various metal-, metal oxide-, and metal sulfide-based antibacterial nanomaterials have been designed to eradicate bacteria through complex mechanisms, including cell membrane disruption, protein inactivation, oxidative stress induction, electrolyte imbalance, and gene expression alterations [20]; however, metal ion-associated cytotoxicity and environmental safety are major concerns [21]. Thus, these are far from replacing traditional antibiotics in animal feed.

Recently, carbon-based nanomaterials, such as carbon nanotubes, graphene oxide, and carbon quantum dots (CQDs), have been demonstrated to display efficient antimicrobial activity and low toxicity [22, 23]. For example, we have previously reported the synthesis of antimicrobial Spd-CQDs made from a biogenic polyamine, spermidine, by a one-step pyrolysis procedure. These effectively treated *Staphylococcus aureus*-induced bacterial keratitis in rabbits [24]. Unlike spermidine alone, these as-prepared Spd-CQDs exhibit broad-spectrum antibacterial activity, mainly owing to enhanced interactions with bacteria via their strong positive charge and the self-preserved pyrolytic products on the surface of the CQDs. In another study, CQDs derived from a mixture of biogenic spermidine and dopamine exhibited highly adhesive properties, which enabled them to prevent biofilm formation on the surface of contact lenses [25]. Additionally, CQDs can exhibit antiviral properties; for example, curcumin-based CQDs improve the water solubility of curcumin and significantly decrease the mortality rate of newborn mice infected with enterovirus 71 [26]. In a challenge trial, polyamine-derived CQDs, added to pellet feed, promoted immune activation in whiteleg shrimp, thereby helping them resist white spot syndrome virus (WSSV) infection [27].

**Fig. 1:**
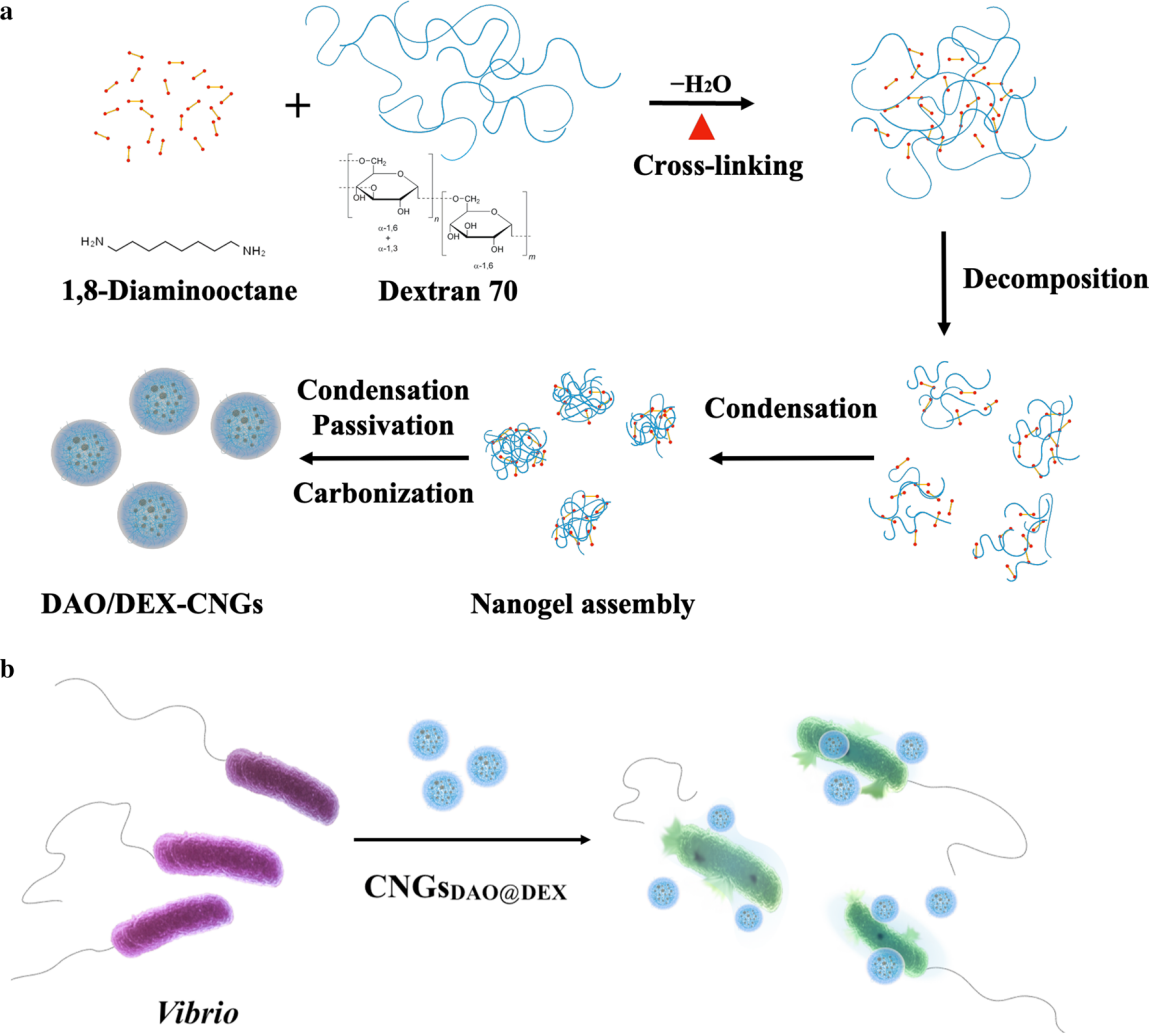
Preparation of antimicrobial CNGs. Schematic representation of **A** the synthesis of multifunctional DAO/DEX-CNGs and **B** their application for combating marine *Vibrio* bacteria

In this study, we continue to explore the potential of carbon-based therapeutic nanomaterials to solve difficult infectious diseases, such as AHPND. A multifunctional carbonized nanogels (CNGs) were prepared via the mild pyrolysis of 1,8-diaminooctane (DAO) and dextran 70 (DEX), named DAO/DEX-CNGs (Fig. 1A). Detail investigation were conducted to support the use of DAO/DEX-CNGs is able to prevent AHPND (Fig. 1B) and reduce antibiotic overuse in shrimp aquaculture.

## Results

### One-step synthesis and characterization of CNGs

Glass vials containing DAO (20 mg), DEX (100 mg), or a mixture of DAO and DEX with mass ratios (DAO:DEX) of 1.0:0.5 (20:10 mg), 1.0:1.5 (20:30 mg), or 1.0:5.0 (20:100 mg) were separately heated at 180 ℃ for 3 h to obtain CNGs. After heating, DAO formed a sticky brown film with poor water solubility (Additional file 1: Fig. S1) as oxidation of the amine groups and polymerization of DAO following carbonization during heating led to the formation of a nitrogen-doped polymeric graphene film with poor hydrophilicity. In contrast, after heating and dissolution in water, DEX yielded a transparent product, probably because of the lower degree of carbonization. The pyrolytic products obtained from the various mixtures of DAO and DEX were brown in color and exhibited high aqueous solubility. Moreover, the solubility of the product increased with an increasing ratio of DEX, owing to its intrinsic high hydrophilicity.

**Fig. 2:**
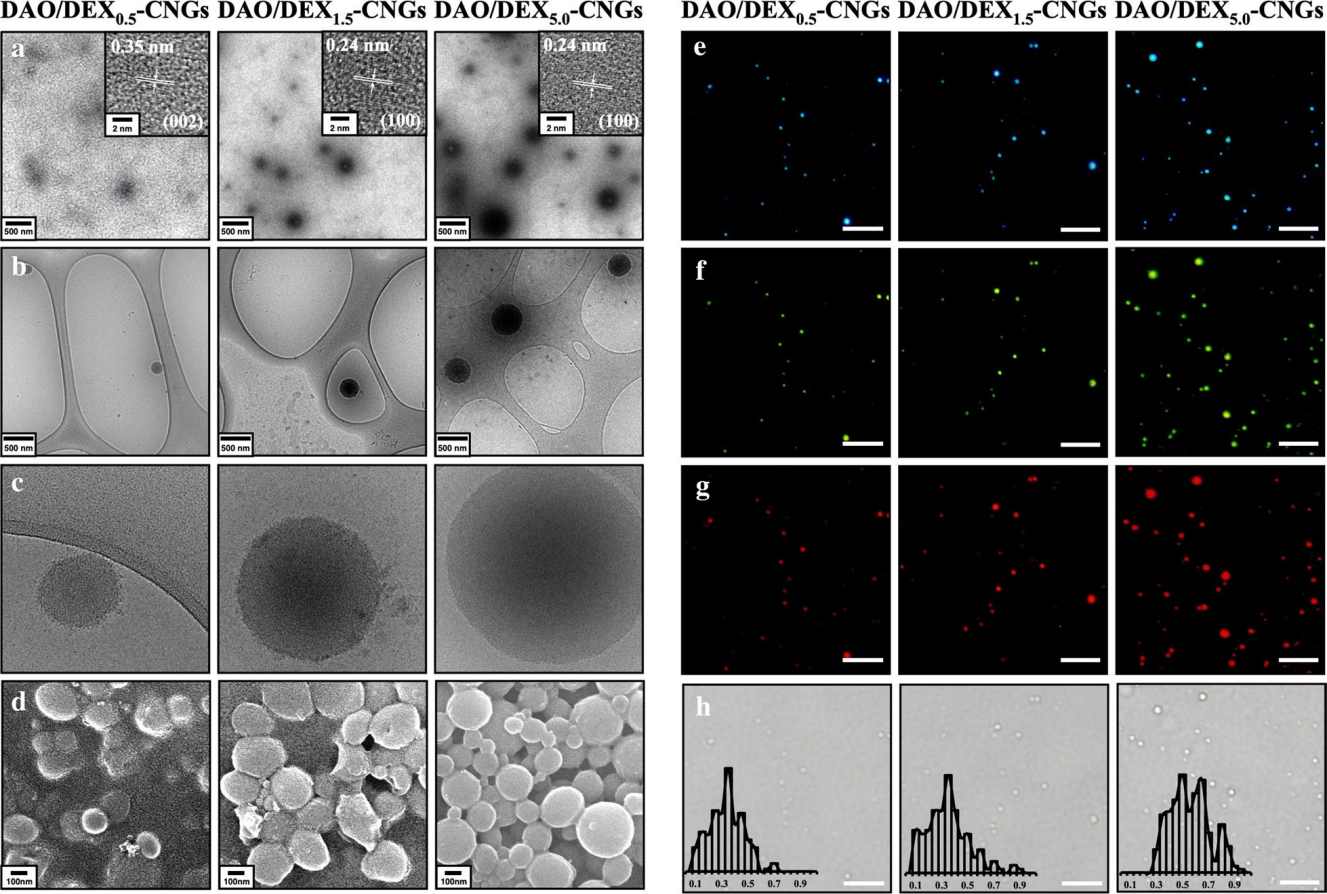
Characterization of DAO/DEX-CNGs. **A** TEM, **B**, **C** cryo-EM, **D** SEM, **E**–**G** fluorescence images, and **H** optical bright-field of DAO/DEX-CNGs. The inset to **A**: HRTEM images of corresponding DAO/DEX-CNGs. Inset in **H**: size distribution histograms of corresponding DAO/DEX-CNGs. Fluorescence images of DAO/DEX-CNGs were recorded at excitation wavelengths of **E** UV (330–385 nm), **F** blue (450–480 nm), and **G** green (510–550 nm). The scale bars in **E** to **H** represent 10 μm

We next characterized the size of CNGs. Transmission electron microscopy (TEM) revealed that, after heating, the mixtures of DAO and DEX formed nanogel structures of 100–750 nm in diameter and polymeric frameworks on the surface of the nanogels (Fig. 2A). For simplicity, these as-prepared nanogels are denoted DAO/DEX_0.5_-CNGs, DAO/DEX_1.5_-CNGs, and DAO/DEX_5.0_-CNGs, based on mass ratios (DAO:DEX) of 1.0:0.5, 1.0:1.5, and 1.0:5.0, respectively, as used in the mixture. The as-prepared DAO/DEX_5.0_-CNGs exhibited the highest product yield (*ca*. 71%), mainly owing to high water solubility (Additional file 1: Table S1). Furthermore, cryogenic electron microscopy (cryo-EM) and scanning electron microscopy (SEM) revealed the presence of spherical CNGs (Fig. 2B–D). Moreover, the lattice structure in the interior of CNGs is discernible in the high-resolution TEM images (Fig. 2A, inset) and by their distinctive fluorescence properties (Fig. 2E–G) that confirm the formation of ultrasmall few-layered graphene quantum dots within as-formed CNGs [28]. The hydrodynamic diameter/ζ-potential of the as-prepared DAO/DEX_0.5_-CNGs, DAO/DEX_1.5_-CNGs, and DAO/DEX_5.0_-CNGs determined by dynamic light scattering were 209 nm/13.2 mV, 322 nm/6.1 mV, and 580 nm/5.8 mV, respectively (Fig. 2H). The higher ratio of polymeric and neutrally charged DEX in DAO/DEX-CNGs resulted in a larger size and smaller charge.

Next, we characterized the spectral properties of CNGs. The UV–visible absorption spectra of all as-prepared CNGs showed broad bands around 230–450 nm (Additional file 1: Fig. S2A). The absorption band around 250–290 nm was attributed to the *π* → π* transition of aromatic/alkenyl C=C bonds or C=N bonds, supporting the formation of graphitic carbon clusters, whereas the shoulder band at around 300–380 nm was probably due to *n* → π* transitions of C=O and C=N bonds [29]. All DAO/DEX-CNGs displayed similar emission maxima profiles around 460 nm when excited at 365 nm (Additional file 1: Fig. S2B). Additionally, the DAO/DEX-CNGs exhibited excitation wavelength-dependent fluorescence emission properties (Fig. 2F–H and Additional file 1: Fig. S2C), mainly owing to the formation of different sizes of polycyclic aromatic or graphene clusters [30]. However, partially carbonized CNGs retained many emissive traps, which lowered their quantum yield (QY) to < 1% (at excitation/emission maxima of 365 and 460 nm; in comparison with the quinine sulfate reference) (Additional file 1: Table S1) [29].

### Synthetic mechanism of CNGs

Some characteristics of the DAO/DEX-CNGs resembled those of small-sized carbon dots (CDs; < 10 nm), such as their excitation wavelength-dependent fluorescence emission. However, the large size of the DAO/DEX-CNGs (> 100 nm) prevented their classification as typical CDs. The CNGs comprised a cross-linked polymer (gel) structure with abundant functional groups and were embedded with graphene-like CQDs. X-ray photoelectron spectroscopy (XPS; Additional file 1: Fig. S3) and Fourier transform infrared spectroscopy (FT-IR; Additional file 1: Fig. S4 and Table S2) demonstrated the presence of diverse functional groups in the CNGs, including O–H, N–H, C–O, C=O, C–N, C=C, and C=N. Furthermore, several functional groups from the precursors DAO and DEX (e.g., O–H, N–H, C–O, and C–N) were preserved. The presence of C=C and C=N in the CNG suggests the formation of aromatic rings and that the nitrogen atoms were incorporated (doped) as pyridinyl, pyrrolyl, and amide moieties in the heterocyclic ring systems [31].

Figure 1A shows the proposed mechanism of formation of the CNGs. Time-course TEM measurements revealed that large irregular gel-like structures formed within 5 min through the crosslinking reaction of DAO and DEX at 180 ℃ (Additional file 1: Fig. S5). During the heating process, the primary amino group within DAO at both terminals acted as a crosslinking agent for DEX polysaccharides to form inter- and intra-crosslinking polymers (or supramolecules) with micrometer sizes through dehydration. The dehydration reaction between the aldehyde groups of the DEX and the amino group of DAO may have resulted in the formation of a Schiff base, followed by rearrangement to form the Amadori product [32]. Then, during the 5–10 min of the pyrolysis reaction, the supramolecular structures partially decomposed into smaller fragments. Further heating produced the smaller and semi-spherical nanostructures through the condensation reaction while in situ partial carbonization occurred. During the subsequent period of heating (1–3 h), spherical nano-colloidal structures formed as a result of further condensation and carbonization. We observed that the as-formed CNGs still featured polymeric frame structures on their surfaces (Additional file 1: Fig. S5). Large-sized carbon particles (> 1 μm) with poor aqueous solubility (< 10 μg mL^−1^) were obtained upon overheating (⁓ 4 h), probably owing to extreme pyrolysis; thus, we limited heating to 3 h. We also compared DEX mixed with different linear alkyl diamines (NH_2_(CH_2_)_*n*_NH_2_; *n* = 2, 4, 6, 8, 10, 12) to prepare CNGs in our current work. It is interesting to note that CNGs were not formed with NH_2_(CH_2_)_*n*_NH_2_ (*n* < 6) and the size of the CNGs was controlled by changing the lengths of the alkyl diamines (*n* ≥ 6) as well as the molecular weight of DEX.

### Antibacterial activities of DAO/DEX-CNGs

We first tested the antibacterial potency of DAO/DEX-CNGs against four strains of non-resistant bacteria (*Escherichia coli*, *S. aureus*, *Pseudomonas aeruginosa*, and *Salmonella enteritidis*) and one strain of multidrug-resistant bacteria (methicillin-resistant *S. aureus*, MRSA). Our previously reported antibacterial CQDs prepared from spermidine (Spd-CQDs; diameter of *ca.* 6 nm) with a high zeta potential (ζ = + 45 mV) exhibited effective antibacterial ability only in low ionic strength solution (5 mM sodium phosphate, pH 7.4) (Additional file 1: Fig. S6) [24]. In contrast, the DAO/DEX_5.0_-CNGs reported herein displayed potent antibacterial activity on all tested bacteria, even in high ionic strength solution, such as phosphate-buffered saline (PBS). Unlike DAO/DEX-CNGs, the Spd-CQDs tend to aggregate and then precipitate in PBS solution owing to electrostatic screening. Among the tested mass ratios, the DAO/DEX_5.0_-CNGs displayed superior bacteriostatic activity to the other DAO/DEX-CNGs in PBS solution. We attribute this to the polymeric features of the large-sized DAO/DEX_5.0_-CNGs exerting strong interaction effects with bacteria despite charge screening in the high ionic strength solution. Indeed, we observed that the DAO/DEX_5.0_-CNGs featured a Velcro-like property, whereby they rapidly bound to *E. coli* and *S. aureus* membranes after only 1 min of incubation in PBS solution (Additional file 1: Fig. S7).

**Fig. 3:**
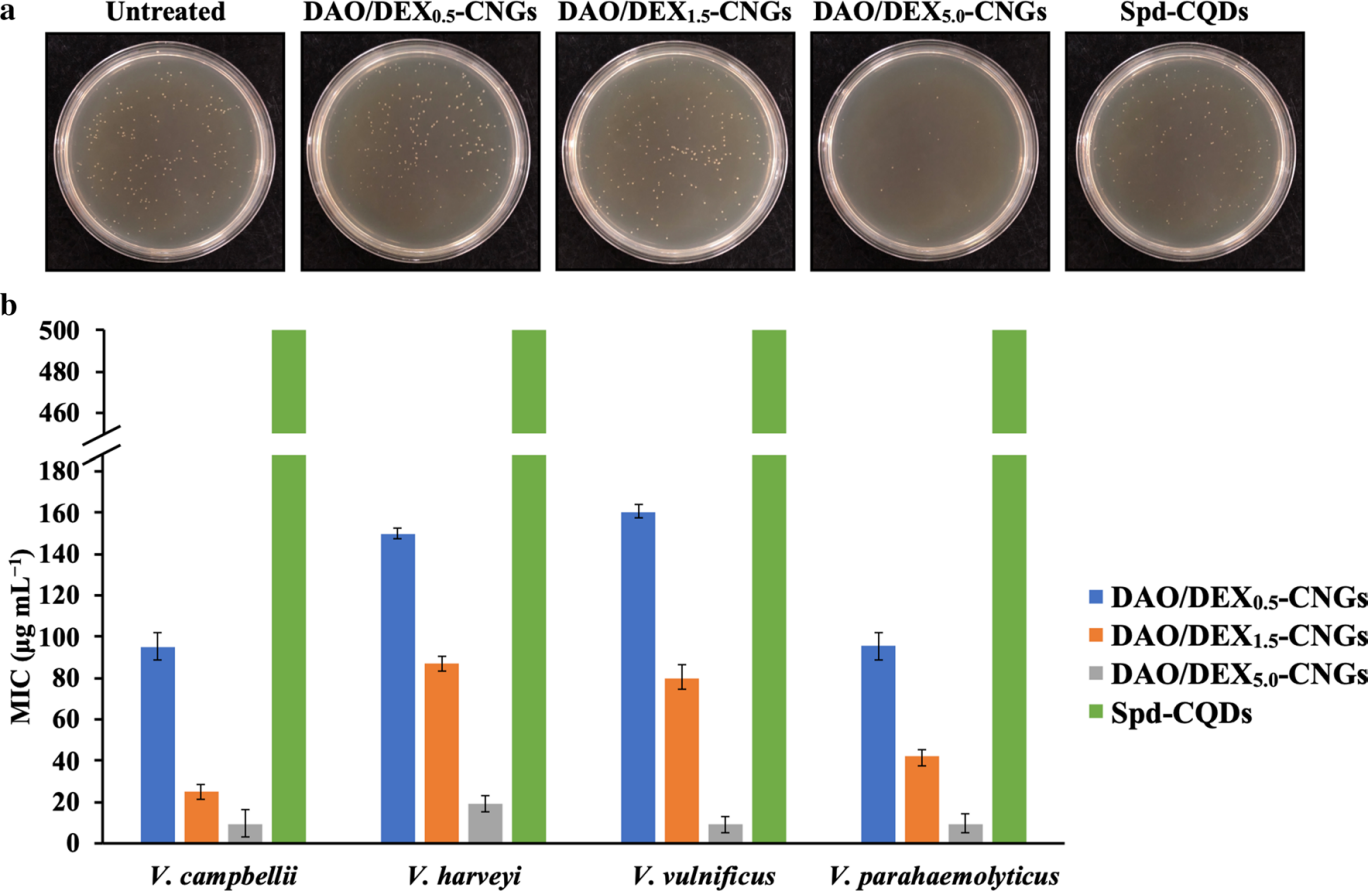
Antibacterial activity of DAO/DEX-CNGs against *Vibrio*. **A** Representative colony formation of *V. parahaemolyticus* untreated or treated with 10 μg mL^−1^ of DAO/DEX-CNGs or Spd-CQDs in artificial seawater. **B** Comparison of the minimum inhibitory concentration (MIC) of DAO/DEX-CNGs and Spd-CQDs in seawater. Error bars represent the standard deviation of three repeated measurements

After demonstrating their superior antibacterial activity toward common pathogenic bacteria, we explored the antimicrobial action of DAO/DEX_5.0_-CNGs toward marine *Vibrio*. Figure 3A displays the results of colony formation assays for *V. parahaemolyticus* that were untreated or treated with DAO/DEX-CNGs or Spd-CQDs in artificial seawater (480 mM NaCl, 27 mM MgCl_2_, 30 mM MgSO_4_, 10 mM CaCl_2_, 10 mM KCl, and 2.0 mM NaHCO_3_). The DAO/DEX_5.0_-CNG-treated group showed > 95% inhibition of the bacteria. The minimal inhibitory concentration (MIC) of DAO/DEX_5.0_-CNGs (9–19 μg mL^−1^) for the tested *Vibrio* strains was much lower than that of DAO/DEX_0.5_-CNGs (95–160 μg mL^−1^), DAO/DEX_1.5_-CNGs (25–87 μg mL^−1^), and Spd-CQDs (> 500 μg mL^−1^) (Fig. 3B). Furthermore, the precursors (i.e., DAO and DEX) exhibited negligible antibacterial activity against representative bacteria (MIC > 1.0 mg mL^−1^) compared with DAO/DEX_5.0_-CNGs. These results suggest that these DAO/DEX_5.0_-CNGs effectively eradicate *Vibrio* bacteria.

**Fig. 4:**
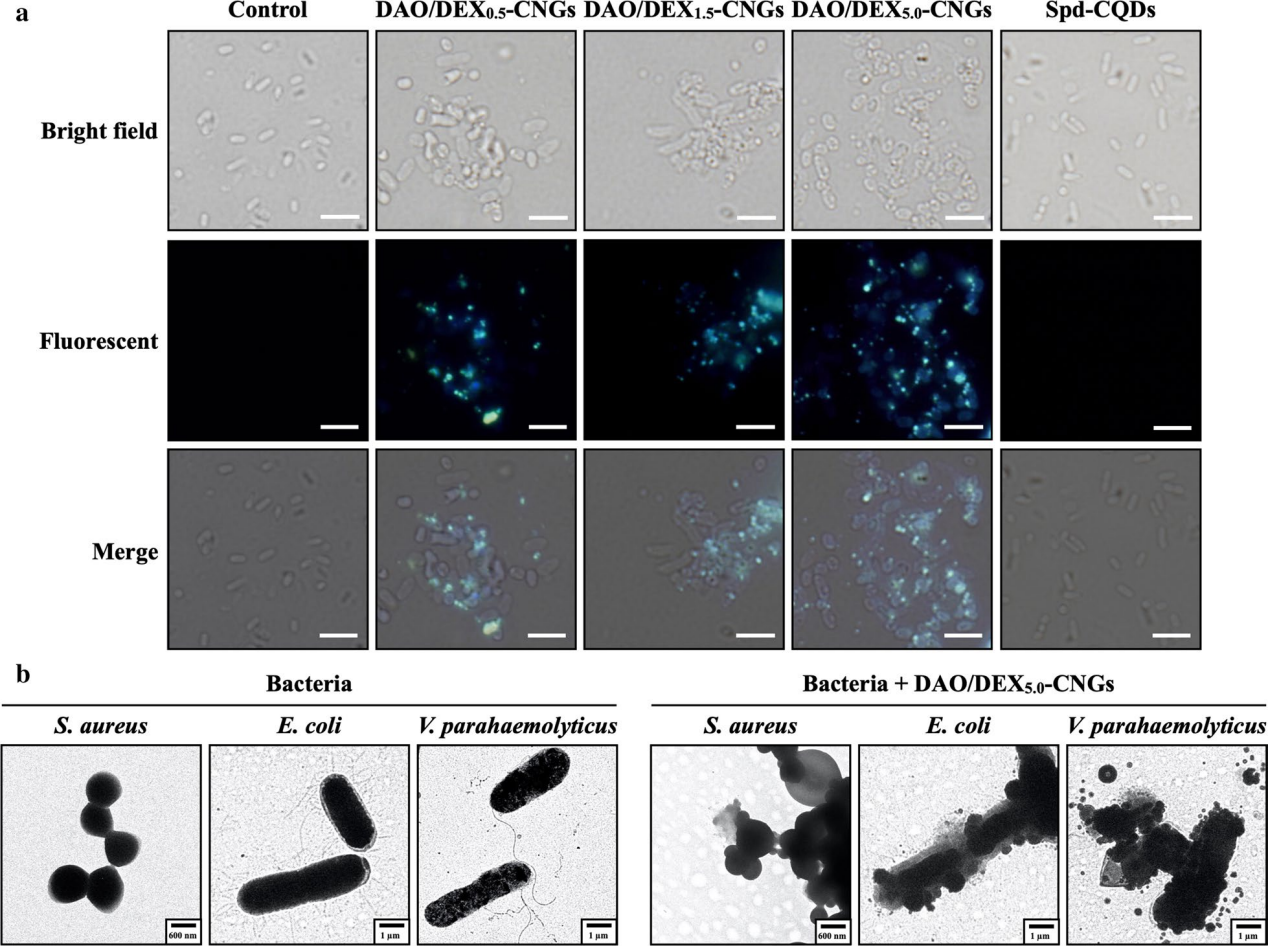
Interaction between DAO/DEX-CNGs and bacteria. **A** Microscopic images of the *V. parahaemolyticus* (1.0 × 10^7^ CFU mL^−1^) incubated without or with 100 μg mL^−1^ of DAO/DEX-CNGs or Spd-CQDs in artificial seawater for 60 min. The fluorescence images in a were recorded using an excitation filter for UV light (360–380 nm). The scale bar represents 10 μm. **B** TEM images of *S. aureus* and *E. coli* in PBS and *V. parahaemolyticus* in artificial seawater in the absence and presence of 100 μg mL^−1^ DAO/DEX_5.0_-CNGs

Microscopic images revealed that the DAO/DEX_5.0_-CNGs, at ~ 500 nm, strongly bound to *V. parahaemolyticus* and led to a higher extent of bacterial aggregation than Spd-CQDs and other DAO/DEX-CNGs (Fig. 4A). Additionally, TEM imaging showed that the as-prepared DAO/DEX_5.0_-CNGs easily deposited onto the bacteria and damaged the integrity of bacterial membranes, thereby causing leakage of the cytoplasm (Fig. 4B).

In the 2′,7′-dichlorodihydrofluorescein diacetate (DCFH-DA) assay, the fluorescent product DCF (*λ*_em_^max^ ≈ 530 nm), which is produced through the sequential reaction of DCFH-DA with cellular esterase and intracellular reactive oxygen species (ROS), was observed in DAO/DEX_5.0_-CNGs-treated *V. parahaemolyticus* (Additional file 1: Fig. S8). ROS generation in DAO/DEX_5.0_-CNG (10 μg mL^−1^)-treated *V. parahaemolyticus* was higher than that in untreated *V. parahaemolyticus* and *V. parahaemolyticus* treated with H_2_O_2_ (10 μg mL^−1^). The catalytic activity of DAO/DEX-CNGs that enables ROS generation arises mainly owing to specific ligands (i.e., C=O and –C–O–C) on the edges of the graphene-like structure [33]. Additionally, the fast electron transportation characteristics of the embedded graphene facilitates ROS generation [31, 33]. Furthermore, nitrogen-doping into the graphene structure as quaternary N and pyridinic N may also contribute to the catalytic formation of ROS [31, 34, 35]. This nitrogen-doping can boost the spin density and charge distribution of carbon atoms, thereby increasing the density of catalytically active centers on the graphene surfaces [31].

### Biocompatibility of DAO/DEX_5.0_-CNGs

In the alamarBlue assay, DAO/DEX_5.0_-CNGs did not show significant cytotoxicity toward any tested cell line up to 100 μg mL^−1^, which was > fivefold higher than the MIC values of the bacteria (Additional file 1: Fig. S9A). Moreover, DAO/DEX_5.0_-CNGs exhibited negligible hemolysis up to 100 μg mL^−1^ (Additional file 1: Fig. S9B).

After 1 week of feeding with commercial feed or DAO/DEX_5.0_-CNGs-mixed feed (1–100 μg g^−1^), even at the highest dose of DAO/DEX_5.0_-CNGs (100 μg g^−1^), the shrimp survival rate remained the same as that in the group fed commercial feed (Additional file 1: Fig. S10A). Thus, our results indicate that DAO/DEX_5.0_-CNGs additives do not cause severe toxicity in shrimp at up to 100 μg g^−1^. Histological results of the hepatopancreas from DAO/DEX_5.0_-CNGs-fed shrimp (up to 100 μg g^−1^) were the same as those from the control group. The hepatopancreas tissue slice samples exhibited a well-organized glandular tubular structure (T), including a star-shaped tubule lumen (Lum) lining with a single layer of normal epithelial cells and Blasenzellen cells (B-cells) with large apical secretory granules (Additional file 1: Fig. S10B). Thus, we have demonstrated that DAO/DEX_5.0_-CNGs is a highly biocompatible material for both human cells and shrimp. Bacterial cell membranes are composed of phospholipid bilayer and are severely affected by the nanomaterial-induced ROS. In contrast, animal cell membranes have less net charge and are rich in cholesterol to strengthen membrane integrity, and hence are less susceptible to the positively charged DAO/DEX_5.0_-CNGs. In addition, animal cells possess various cellular antioxidant enzymes, which can regulate the cellular ROS [36–39]. Similarly, the antioxidant-related enzymes in shrimp cells, such as superoxide dismutase (SOD), catalase (CAT), and glutathione peroxidase (GPx) can avoid damage caused by oxidative stress [40–43], implying that DAO/DEX5.0-CNGs is a safe feed additive for shrimp aquaculture.

### DAO/DEX_5.0_-CNGs protect shrimp from *Vibrio* infection

**Fig. 5:**
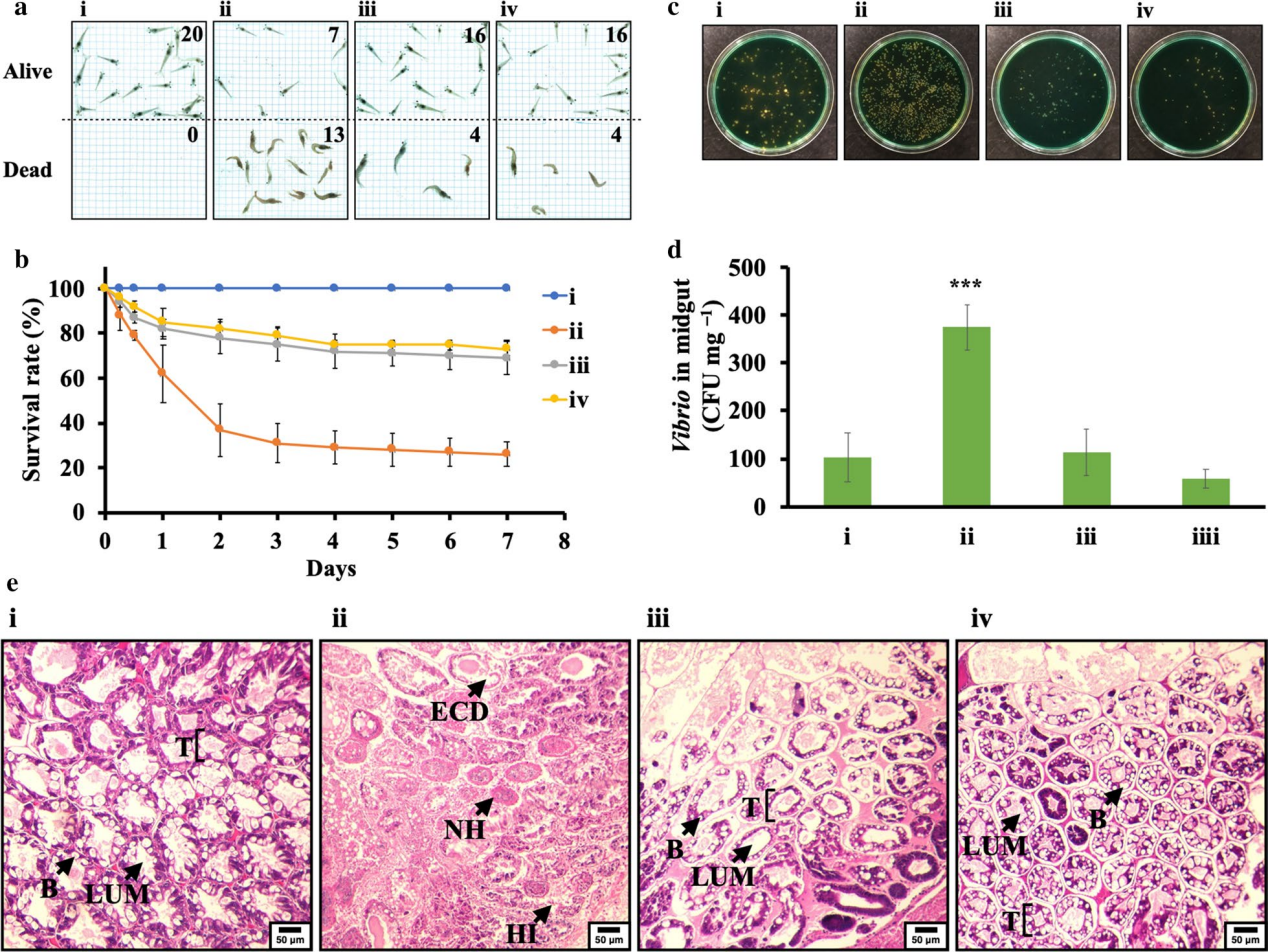
DAO/DEX_5.0_-CNGs as a potential feed additive. **A** Photographs of (i) non-infected (negative control) and (ii to iv) *V. parahaemolyticus*-infected shrimp after feeding with (ii) commercial feed or feed mixed with (iii) 10 μg g^−1^ and (iv) 100 μg g^−1^ of DAO/DEX_5.0_-CNGs for 3 days. **B** Survival rate (%) vs time (days) for corresponding shrimp groups. **C** Representative colony formation assays and **D** quantification of *V. parahaemolyticus* isolated from the midgut of corresponding shrimp at day 1. **E** Histopathological analysis of hepatopancreas at day 3. Epithelial cell detachment (ECD); Hemocytic nodules (HN); Hemocytic infiltration (HI); Tubule (T); Lumen (Lum); Blasenzellen (B-cells, **B**). Error bars in (**B**) and (**D**) represent the standard deviation of three repeated measurements. Asterisks indicate statistically significant differences (***p < 0.001; *n* = 3) compared to the control groups on day 1

After seven days of feeding with commercial feed or the feed mixed with 10 or 100 μg g^−1^ DAO/DEX_5.0_-CNGs and *V. parahaemolyticus* challenge, infected shrimp displayed typical pathological signs, including lethargy, empty gut, paleness, and aqueous hepatopancreas, in the control group (i.e., feed without DAO/DEX_5.0_-CNGs additive) [34]. In contrast, only mild signs were observed in shrimp fed DAO/DEX_5.0_-CNGs. Additionally, the survival rates on day 7 post-challenge were significantly improved (*p* < 0.001) in the DAO/DEX_5.0_-CNGs fed groups compared with those in the control group (Fig. 5A, B). Specifically, the survival rate increased from 26% when fed with commercial feed to 73% when the feed was mixed with 100 μg g^−1^ DAO/DEX_5.0_-CNGs.

Pathogenic *Vibrio* easily colonizes the digestive system, where it starts to cause diseases [44]. We hypothesized that the protective effects of antibacterial DAO/DEX_5.0_-CNGs may arise via the suppression of the colonization of *V. parahaemolyticus* in the intestinal organs. Therefore, we collected midgut tissues from challenged shrimp to evaluate the in vivo antimicrobial potency of DAO/DEX_5.0_-CNGs. After sequential tissue homogenization, serial dilutions, and plating, the total number of *Vibrio* colonies in each sample was counted on thiosulfate citrate bile salts sucrose (TCBS) agar plates. In the control group, wherein shrimp were fed with commercial feed without *Vibrio* challenge, *Vibrio* was detected in the digestive canal (Fig. 5C(i) and D(i)); however, the number of *Vibrio* colonies increased significantly after *Vibrio* challenge (Fig. 5C(ii) and D(ii)). In shrimp that ingested DAO/DEX_5.0_-CNGs, the number of *Vibrio* colonies was even lower than that in the control group when 100 μg g^−1^ DAO/DEX_5.0_-CNGs was applied (Fig. 5C(iv) and D(iv)). Our results indicate that DAO/DEX_5.0_-CNGs exert a superior antibacterial capability, compared to previous antibacterial compounds which exert robust antibacterial effects, even in the gut of shrimp, thereby reducing *Vibrio* infection.

Although the number of *Vibrio* colonies in the midgut of whiteleg shrimp fed 10 μg g^−1^ DAO/DEX_5.0_-CNGs was higher than that in shrimp fed 100 μg g^−1^ DAO/DEX_5.0_-CNGs (Fig. 5C, D), the survival rates were similar between groups (Fig. 5B). We, therefore, analyzed hepatopancreas tissue using pathological methods. In the hepatopancreas tissue slides of shrimp challenged with *V. parahaemolyticus*, we observed typical pathological features of AHPND, including pigment loss in the connective tissue capsule, irregular shape of tubal lumen with epithelial cell detachment, loss of secretory granule in B-cells, hemocytic nodules, and hemocytic infiltration (HI) (Fig. 5E) [45]. When the shrimp were fed with DAO/DEX_5.0_-CNGs additives, their hepatopancreas exhibited relatively mild signs of AHPND. Moreover, the lower dose of DAO/DEX_5.0_-CNGs (10 μg g^−1^) still reduced the histopathological damage and HI compared with the control group (without DAO/DEX_5.0_-CNGs). These results reveal that the DAO/DEX_5.0_-CNGs may have functions, other than bactericidal effects, that protect shrimp from AHPND.

### Immunomodulation of DAO/DEX_5.0_-CNGs in shrimps

Our previous work demonstrated that Spd-CQDs stimulate several immune-related genes, including lysozyme, anti-lipopolysaccharide factor, and cytosolic manganese superoxide dismutase genes, that prevent WSSV infection [27]. Many studies have also reported that nanomaterials regulate the immune systems of animals [46–48]. Therefore, we further evaluated the shrimp immune system after treatment with DAO/DEX_5.0_-CNGs to elicit their contribution toward immune protection. After feeding shrimp in the presence or absence of DAO/DEX_5.0_-CNGs (10 or 100 μg g^−1^) for three days, their hemocytes were collected, and the expression levels of immune-related genes including *β*-1,3-glucan-binding protein (LGBP), anti-lipopolysaccharide factor (ALF), lysozyme (LYZ), and cytosolic manganese superoxide dismutase (cytMnSOD), were analyzed by real-time quantitative reverse-transcription PCR (qRT-PCR). These genes represent either important sensors or effecters in the shrimp immune system against various bacterial pathogens [49–52]. Feeding shrimp DAO/DEX_5.0_-CNGs did not increase the expression of *LGBP*, *ALF*, or *LYZ*, and that of *cytMnSOD* was slightly decreased (Additional file 1: Fig. S11). Unlike the other genes, *cytMnSOD* is not only a responder when the immune system is challenged but is also sensitive toward exposure to toxic materials [52]. We suggest, therefore, that DAO/DEX_5.0_-CNGs does not act as an immune stimulator in the shrimp. Instead, the decrease in *cytMnSOD* expression might be explained by the antibacterial activity of DAO/DEX_5.0_-CNGs, which may reduce the gut microbial population and their toxin production.

In the hemolymph, the expression of *LGBP*, *ALF*, *LYZ*, and *cytMnSOD* increased sharply within 24 h after *V. parahaemolyticus* challenge in shrimp fed normal feed (Additional file 1: Fig. S11). Such an acute and severe bacterial infection might cause systemic immune failure, also known as sepsis. The overstimulated immune system and oxidative stress during sepsis usually damage tissues and cause mortality, often to the point that even extensive antibiotic administration is unable to facilitate recovery [53]. Although the pathological mechanism of shrimp sepsis remains unclear, we did observe similar signs, such as overstimulation of the immune system in severe *V. parahaemolyticus* infection. The expression levels of the four immune-related genes were significantly reduced in shrimp fed DAO/DEX_5.0_-CNGs; those of *LGBP* and *cytMnSOD* even returned to that of the unchallenged condition (Additional file 1: Fig. S11). In addition to its antibacterial activity, immune inhibition by DAO/DEX_5.0_-CNGs might contribute to the higher survival rate of shrimp after acute and severe *V. parahaemolyticus* infection.

### DAO/DEX_5.0_-CNGs sponge PirAB toxin

Although DAO/DEX_5.0_-CNGs reduced colonization by *V. parahaemolyticus* in the intestine of whiteleg shrimp, the PirAB toxin remains harmful to the hepatopancreas and lethal to the shrimp. Previous work has shown that the mortality of PirAB toxin-challenged shrimp increases with *ALF* knockdown [54]. ALF is a short antimicrobial polypeptide that binds to lipopolysaccharides and peptidoglycans from *V. parahaemolyticus* to alleviate AHPND [55]. Molecular modeling and docking studies have revealed that the lipopolysaccharide-binding sites of ALF also interact with PirB, thereby reducing PirAB toxicity [54]. The expression of ALF was significantly reduced in shrimp fed DAO/DEX_5.0_-CNGs followed by *V. parahaemolyticus* challenge, compared to the controls. Thus, DAO/DEX_5.0_-CNGs might act as a sponge to absorb the PirAB toxin through their polymeric nature and compensate for the need for ALF. To test this, we prepared recombinant PirA (0.1 mg) or PirB (0.1 mg) toxins and incubated them with different amounts of DAO/DEX_5.0_-CNGs (0.5–10 mg). After removing absorbed PirA or PirB by centrifugation, the supernatants were analyzed by western blotting. The DAO/DEX_5.0_-CNGs (5.0 mg) adsorbed > 80% of PirA (0.1 mg) and PirB (0.1 mg) toxin (Additional file 1: Fig. S12). Based on these observations, we believe that highly efficient trapping of lethal PirA/B toxins by DAO/DEX_5.0_-CNGs also contributes toward their excellent protective effects for whiteleg shrimp against AHPND.

### Effects of DAO/DEX_5.0_-CNGs on the microbiota of shrimp

It is now recognized that the gut microbiota plays indispensable roles in several key physiological functions of shrimp [56]. The intestinal bacterial composition and their metabolites may highly affect nutrient acquisition and susceptibility to pathogenesis in shrimp [57]. Antibacterial agents that impact the gut microbiota may also affect shrimp. Therefore, different doses of DAO/DEX_5.0_-CNGs (0, 10, and 100 μg g^−1^) were fed to shrimp for 7 days, after which their gut microbiota was analyzed by a standard Illumina 16S rRNA gene amplicon sequencing method. The prokaryotic populations in each sample were analyzed using tag-encoded high-throughput sequencing of the V3–V4 region of 16S rRNA gene amplicons. In total, 1,277,642 high-quality sequences were obtained, with an average number of 70,980 reads (ranging from 28,131 to 176,016 reads per sample). The read dataset of each library was randomly subsampled to ensure an even sampling depth (28,131 reads per library). In total, 4432 operational taxonomic units (OTUs) were obtained; the number of OTUs detected in each sample was 138–316, with an average of 246.

Similarly to previous reports [58, 59], the relative abundance of OTUs at the phylum level showed that the microbiota of control diet whiteleg shrimp was mainly composed of Proteobacteria (54% ± 3%) and Bacteroidetes (36% ± 11%) (Additional file 1: Fig. S13; S1 group). After 4 days of feeding with DAO/DEX_5.0_-CNGs additives (10 μg g^−1^), Proteobacteria (39% ± 3%) and Bacteroidetes (51% ± 2%) remained the dominant phyla in the shrimp intestines (S2 group). Upon feeding with a higher dose of DAO/DEX_5.0_-CNGs (100 μg g^−1^), the relative abundance of Proteobacteria (43% ± 4%) and Bacteroidetes (41% ± 8%) was slightly changed, but they still dominated.

**Fig. 6:**
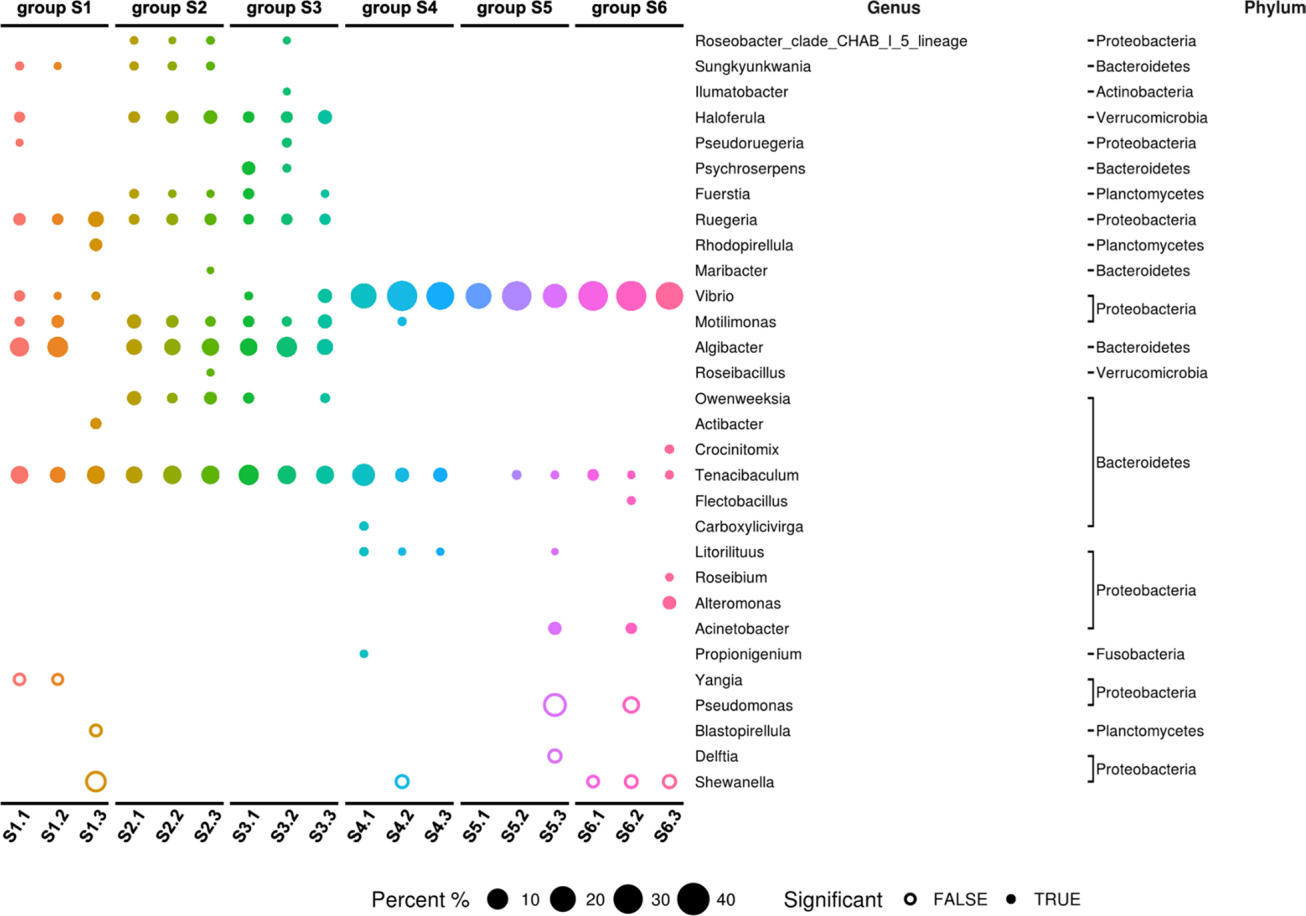
Distribution of sequence read abundance of bacterial populations in each sample of shrimp midgut. (S1 to S3) non-infected and fed commercial feed (negative control) (S1) or feed mixed with 10 μg g^−1^ (S2) and 100 μg g^−1^ (S3) of DAO/DEX_5.0_-CNGs for 3 days; (S4 to S6) *V. parahaemolyticus* infected shrimp after feeding with commercial feed (S4) or feed mixed with 10 μg g^−1^ (S5) and 100 μg g^−1^ (S6) of DAO/DEX_5.0_-CNGs for three days. The bacterial phylum and genus are displayed on the right side of the figure

Next, we analyzed samples from each group individually. We selected species (i.e., OTUs) that had an abundance greater than 1% and appeared in at least one of the samples. The top 30 species were then selected according to their ranking via their *p*-values from the two-tail Wilcoxon rank-sum test or Kruskal–Wallis test [60]. There were some individual differences in the top 30 species, even within the same experimental groups (Fig. 6; S1-1, S1-2, and S1-3). However, feeding with DAO/DEX_5.0_-CNGs resulted in no significant difference in the abundance of the dominant bacteria, including *Tenacibaculum*, *Alglbacter*, *Motilimonas*, and *Ruegeria*, compared with that in the control diet group. It is also worth noting that *Vibrio* did not reach the 1% abundance threshold in three successive measurements of the S2 group. However, in S3-1 and S3-3, which were fed with higher concentrations of DAO/DEX_5.0_-CNGs, *Vibrio* still remained at 1.22% and 4.84% abundance, respectively.

Using t-distributed stochastic neighbor embedding (t-SNE) analysis to visualize the differences between each data set, samples in group S1–S3 were closely clustered (Additional file 1: Fig. S14). Certain individual variations, such as between S1-1 and S1-3, were even higher within the same group than in the intergroup comparison. Overall, DAO/DEX_5.0_-CNGs did not exhibit a strong impact on the dominant species in the shrimp gut microflora, including *Vibrio*. This is consistent with our observations that DAO/DEX_5.0_-CNGs did not result in a significant difference in antimicrobial activities of various bacteria (Fig. 3B and Additional file 1: Fig. S6).

Previous reports have shown that feeding with an antibiotic, such as ciprofloxacin or sulfonamide, causes a significant decrease (> 50%) in shrimp intestinal OTUs, and the Shannon index analysis indicated that using antibiotics also decreases the diversity of the intestinal microflora [59]. However, in our case, there was no statistical difference in OTUs nor in the Shannon index in each group, including the S3 group which had been fed with the highest dosage of DAO/DEX_5.0_-CNGs (100 μg g^−1^) (Additional file 1: Fig. S15). Because of the non-selective bactericidal effect of DAO/DEX_5.0_-CNGs, the levels of major species and minor species were evenly decreased and preserved. Compared with antibiotics, it might be an add up for employing as a feed additive in shrimp.

When shrimp were challenged by AHPND-causing *V. parahaemolyticus*, the intestinal microflora was greatly influenced both at the phylum (Additional file 1: Fig. S13A) or the genus level (Additional file 1: Fig. S13B), even in the DAO/DEX_5.0_-CNGs fed groups. In general, the abundance of Proteobacteria increased significantly in all challenged groups, particularly those from the genus *Vibrio*. The t-SNE analysis also showed that the bacterial composition after *Vibrio* challenge was very different from the healthy groups (Additional file 1: Fig. S14). This phenomenon during an AHPND outbreak is named dysbiosis of the gut [44]. However, even in the DAO/DEX_5.0_-CNGs treated groups, the intestinal microflora could not be restored to the state of the healthy groups. Owing to their non-selective nature, DAO/DEX_5.0_-CNGs could only reduce the absolute number of pathogens (Fig. 5B). A possible explanation is that our experimental shrimp were kept in a water tank containing a high dose of *V. parahaemolyticus*, even after challenging. Pathogenic *Vibrio* in the culture environment may continue to affect the shrimp gut ecosystem via the oral or anal routes, and in turn, *Vibrio* remained dominant in the intestinal microflora. Additionally, even if *Vibrio* had been eliminated by DAO/DEX_5.0_-CNGs, its DNA may have still been included in the OTU count [61]. These are in agreement with the fact that after feeding DAO/DEX_5.0_-CNGs, the number of viable *Vibrio* colonies did decrease significantly (Fig. 5C) and the survival rate of whiteleg shrimp was also greatly improved.

We further investigated the accumulation of DAO/DEX_5.0_-CNGs in shrimp by tagging with rhodamine B isothiocyanate (RITC) (Additional file 1: Fig. S16). First, DAO/DEX_5.0_-CNGs was functionalized with RITC, which showed uninterrupted fluorescence properties. Subsequently, the RITC-derived DAO/DEX_5.0_-CNGs were mixed with the commercial shrimp feed (100 μg g^−1^) and fed the shrimp for 7 days. During the 7-day period, the shrimp did not die or behave abnormally, indicating that DAO/DEX_5.0_-CNGs additives did not cause serious physiological effects. Further, in vivo fluorescence imaging of shrimp after being fed with DAO/DEX_5.0_-CNGs additives for 7 days showed negligible fluorescence signal in the shrimp organs (e.g., stomach, hepatopancreas, gut), indicating that low-dose feeding of DAO/DEX_5.0_-CNGs leads to very little bioaccumulation in the shrimps. We did not study the depuration kinetics of DAO/DEX_5.0_-CNGs since its bioaccumulation is too low to be detected. We believe the very low dose of DAO/DEX_5.0_-CNGs (100 μg g^−1^) in commercial shrimp feed and low bioaccumulation in the shrimps highly reduce the food safety risk to humans.

## Discussion

Currently, there is no effective solution for AHPND. For example, antibiotic administration cannot prevent damage from PirAB toxins and antibiotic resistances genes can be acquired through HGT [17]. Although the efficacy of antibiotics is decreasing, shrimp farmers use more owing to the absence of alternatives. Eventually, antibiotic overuse may lead to the emergence of a superbug. Thus, AHPND continues to hurt the multi-billion-dollar shrimp aquaculture industry and human’s public health as well [7–9].

We were one of the first groups to discover that CQDs could be a new type of nanomedicine [62]. Our Spd-CQD can cure serious bacterial keratitis and has been regarded as a new hope to clean infection [63]. Using pyrolysis, many natural compounds have been converted to carbon-based therapeutic agents [24–27]. This process resembles the decoction techniques used in traditional Chinese herbal medicine to improve, create new functionalities, and retain desirable characteristics of precursor phytochemicals by carbonization [64]. The carbonization of Chinese herbs inspired us to synthesize carbon-based nanomaterials with multiple functions through multiple precursor combinations [65].

It is exciting to observe the rapid progress of this new field [66]. However, more detailed studies of the biosafety and therapeutic mechanisms of CQDs are needed before the large-scale application of these materials. Including this study, many toxicity studies, using model organisms, have shown no adverse effects of these carbon-based nanomaterials on animals after short- and long-term ingestion [67, 68]. Many processed foods consumed daily also contain CQDs [69–71]. Moreover, a human clinic trial of carbon-based nanoparticles has been conducted in China recently [72]. Although it may still take years until these therapeutic carbon-based nanomaterials can be applied in humans, they can be applied as antibiotic alternatives to treat animal diseases sooner.

## Conclusions

Here, we conducted a comprehensive safety evaluation of DAO/DEX_5.0_-CNGs in whiteleg shrimp to facilitate the future development of carbon-based nanomedicines. Such multifunctional carbon-based nanomaterials hold great potential as feed-additives to treat AHPND and reduce antibiotic overuse in aquaculture. This study also opens the door for the development of new therapeutic carbon-based materials. Unlike traditional herbal medicine, therapeutic carbon-based nanomaterials are evidence-based and it is known the precursor composition, structure, and functional groups of a carbon-based nanomaterial are closely related to its therapeutic effects. In the future, therefore, by choosing precursor substances, composite conditions, and process optimization, other novel materials with more functions may be designed to treat complex diseases, such as AHPND, that current medicines are unable to treat.

## Methods

### Materials

1,8-Diaminooctane (DAO), spermidine trihydrochloride (Spd), sodium chloride, potassium chloride, magnesium chloride, calcium chloride, sodium dihydrogen phosphate, sodium phosphate dibasic, lysogeny broth (LB), tryptic soy broth (TSB), thiosulfate citrate bile salts sucrose (TCBS), agar, trypan blue, and alamarBlue were purchased from Sigma-Aldrich (St. Louis, MO, USA). Dextran 70 (DEX) was obtained from GE Healthcare (Piscataway, NJ, USA). All cell culture media were acquired from Gibco BRL (Grand Island, NY, USA). Antibiotic–Antimycotic (100×), l-glutamine, and nonessential amino acids (NEAA) were brought from Biowest (Lewes, UK). 2ʹ,7ʹ-dichlorodihydrofluorescein diacetate (DCFH-DA) was purchased from Cayman Chemicals (Ann Arbor, MI, USA). Phosphate-buffered saline (PBS; containing 137 mM NaCl, 2.7 mM KCl, 10 mM Na_2_HPO_4_, and 2.0 mM KH_2_PO_4_; pH 7.4) was used to mimic physiological conditions. Milli-Q ultrapure water (18.2 MΩ cm; EMD Millipore, Billerica, MA, USA) was used in all experiments.

### Synthesis of carbonized nanogels (CNGs)

Glass vials (20 mL) containing 1.0 mL of a mixture of 1,8-diaminooctane (DAO)/Dextran 70 (DEX) in a 20/10, 20/30, or 20/100 mg/mg dry mass ratio dissolved in deionized (DI) water were separately heated in an oven (DH 300, Dengyng, New Taipei City, Taiwan) at 180 ℃ for 3 h. The solid residue obtained after the pyrolysis was cooled to room temperature (25 ℃) and dispersed in 4.0 mL of DI water through sonication for 2 h. Each of the solutions was then centrifuged at a relative centrifugal force (RCF) of 500*g* for 30 min to remove the insoluble residue. The supernatant containing CNGs was stored at 4 ℃ until further use.

### Bacterial cultures and antibacterial assays

*Escherichia coli* (*E. coli*; ATCC 47076), *Pseudomonas aeruginosa* (*P. aeruginosa*; ATCC 27853), *Salmonella enterica* (*S. enterica*; ATCC 35664), *Staphylococcus aureus* (*S. aureus*; ATCC 25923), and methicillin-resistant *Staphylococcus aureus* (MRSA; ATCC 43300) were obtained from American Type Culture Collection (ATCC, Manassas, VA, USA), and were grown in lysogeny broth (LB) medium at 37 ℃. The pathogenic strains of *Vibrio campbellii* (*V. campbellii*; ATCC BAA-1116), *Vibrio harveyi* (*V. harveyi*; ATCC 35084), and *Vibrio vulnificus* (*V. vulnificus*; ATCC 27562) were also acquired from ATCC. *V. parahaemolyticus*; isolated from *Litopenaeus vannamei,* was provided by Prof. Han-Ching Wang from National Cheng Kung University (NCKU, Tainan, Taiwan). All *Vibrio* strains were grown separately in tryptic soy broth (TSB) medium with 3% NaCl at 25 ℃. An individual colony of each strain was lifted from the corresponding agar plates, was inoculated in liquid culture media, and then incubated at room temperature with orbital shaking (200 rpm) until the absorbance at 600 nm (OD_600_) had reached 0.40–0.60 (optical path length: 1.0 cm). Next, 1.0 mL of bacterial suspensions and *Vibrio* strains were centrifuged at 3000*g* at 25 °C for 5 min and washed three times in 5 mM sodium phosphate buffer (pH 7.4, 1.0 mL) and 5 mM sodium phosphate (pH 7.4) containing 3% NaCl, respectively. The minimum inhibitory concentration required to kill > 90% of the bacterial population (MIC) of the CNGs was determined by the microtiter broth dilution method. The CNGs were treated with *Vibrio* or other bacteria (10^4^ CFU mL^−1^) in PBS solution or 5.0 mM sodium phosphate buffer (pH 7.4) at room temperature for 3 h. Then, 100 μL from each suspension (10^4^ CFU mL^−1^) was spread onto the TSB or LB agar plates, and CFUs were counted after incubating at 25 or 37 °C for 24 h.

To evaluate the in vivo antibacterial activity of CNGs in the midgut of shrimp, a similar procedure was employed as mentioned above but with slight modifications. All shrimps were euthanized by immersion in ice for more than 15 min as per the guidelines of the American Veterinary Medical Association (AVMA) [73]. The faeces in the midgut were removed and treated with a homogenizing pestle. Then, the homogenized residue was dissolved in a TSB medium with 3% NaCl and plated onto solidified agar plate for CFU counting.

### Experimental shrimp and *V. parahaemolyticus* challenge

The *Litopenaeus vannamei* shrimp (body-weight; 1.0 ± 0.2 g) were purchased from Taikong Corporation (Taipei, Taiwan). All shrimp were raised in 54 L tanks containing 45 L seawater (*ca.* 36 practical saline units) at 25 ℃. Commercial pellet feed for the shrimp was purchased from the TAI TZI CO., LTD. (Chiayi, Taiwan) and the shrimp were fed twice a day (3% of body-weight) during the adaptation and experimental period. To prepare CNGs as a feed additive, the CNGs (2.0 mL; 1–1000 μg mL^−1^) were separately dissolved in DI water and were homogeneously sprayed on the commercial feed (20 g), mixed well and air dried at room temperature. These modified feeds were stored at − 80 ℃ for further use.

The immune challenge studies were carried out as per the procedure of Tran et al*.* with slight modification [74]. Briefly, the shrimp were divided into different groups (20/group) and fed with the relevant feed for 3 days before *V. parahaemolyticus* infection. The shrimp were immersed in solution containing *V. parahaemolyticus* (100 mL; 10^9^ CFU mL^−1^) and seawater (900 mL) for 15 min to induce infection. The shrimp were then transferred to a new tank (25 L) containing seawater (5 L) and *V. parahaemolyticus* (10^4^ CFU mL^−1^). Each group was fed with commercial feed or CNGs-sprayed feed (3% of body-weight) every 12 h for 7 days. The survival rate of the shrimp was recorded at 6 h, 12 h, and every 24 h thereafter. During this process, the dead shrimps were taken out and stored at − 20 ℃ for photographs.

Refer to the Additional file 1 for the details on the materials, preparation of Spd-CQDs, nanomaterial characterization, bacteria labeling, TEM images for bacteria, DCFH-DA assays, in vitro cytotoxicity assays, hemolysis assays, histopathological analysis of shrimp hepatopancreas, RNA extraction, cDNA synthesis, real-time PCR analysis, shrimp stool sampling, microbiota analysis, *Vibrio parahaemolyticus* toxin recombinant protein, toxin adsorption, and statistical methods.

## Supplementary Information

**Additional file 1.** Additional methods, figures and tables.

## Data Availability

All data generated or analyzed during this study are included in this published article.
