# Supplementary material for: Multifunctional carbonized nanogels to treat lethal acute hepatopancreatic necrosis disease
Source: J Nanobiotechnology. 2021 Dec 24;19:448. doi: 10.1186/s12951-021-01194-8 (PMC8710021; doi:10.1186/s12951-021-01194-8)
Supplement: Supplementary file 1 — Additional file 1. Additional methods, figures and tables. [file 12951_2021_1194_MOESM1_ESM.docx]

**Additional file 1**

**Multifunctional Carbonized Nanogels to Treat Lethal Acute Hepatopancreatic Necrosis Disease**

**Author:** Shao-Chieh Yen^1^, Ju-Yi Mao^1,2^, Hung-Yun Lin^1^, Huai-Ting Huang^3^, Scott G. Harroun^4^, Amit Nain^5^, Huan-Tsung Chang^5^, Han-You Lin^6^, Li-Li Chen^7,8^, Chih-Ching Huang^1,8,9,*^ and Han-Jia Lin^1,8,*^

**Affiliations:**

^1^ Department of Bioscience and Biotechnology, National Taiwan Ocean University, Keelung 202301, Taiwan

^2^ Doctoral Degree Program in Marine Biotechnology, National Taiwan Ocean University, Keelung 202301, Taiwan

^3^ Department of Aquaculture, National Taiwan Ocean University, Keelung 202301, Taiwan

^4^ Department of Chemistry, Université de Montréal, Montréal, Québec H3C 3J7, Canada

^5^ Department of Chemistry, National Taiwan University, Taipei, 10617, Taiwan

^6^ Department of Veterinary Medicine, School of Veterinary Medicine, National Taiwan University, Taipei 10617, Taiwan

^7^ Institute of Marine Biology, National Taiwan Ocean University, Keelung 202301, Taiwan

^8^ Center of Excellence for the Oceans, National Taiwan Ocean University, Keelung 202301, Taiwan

^9^ School of Pharmacy, College of Pharmacy, Kaohsiung Medical University, Kaohsiung 80708, Taiwan

*** Corresponding authors:**

E-mail addresses: huanging@ntou.edu.tw (C.-C. Huang), [hanjia@ntou.edu.tw](mailto:hanjia@ntou.edu.tw) (H.-J. Lin)

**Additional method**

**Synthesis of Spd-CQDs**

The synthesis of spermidine-carbon quantum dots (Spd-CQDs) has been described in our previous study [1]. Briefly, spermidine trihydrochloride (20 mg) was dry heated in an oven at 270 ℃ for 3 h. After pyrolysis, the solid residue was cooled to ambient temperature and then dissolved in DI water (5.0 mL). The solution was then sonicated, centrifuged, and dialyzed to purify Spd-CQDs. The purified solutions of Spd-CQDs were stored at 4 ℃ when not in use.

**Characterization of CNGs**

The hydrodynamic sizes and zeta potential (ζ) of the CNGs were measured by a Zetasizer (Nano ZS, Malvern Instruments, Worcestershire, UK) in 5 mM sodium phosphate buffer (pH 7.4). The fluorescence quantum yield (QY) of the CNGs was determined using quinine sulfate (QY = 54% in 0.1 M H_2_SO_4_) as the standard. The fluorescence and UV−Vis absorption spectra of CNGs were recorded using a monochromatic microplate spectrophotometer (Synergy 4 Multi-Mode; Biotek Instruments, Winooski, VT, USA). Fourier transform infrared (FT-IR) spectra in transmission mode within 500−4,000 cm^−1^ were recorded by the FT-IR-6100 (JASCO, Tokyo, Japan) to identify the possible functional groups in the CNGs. Elemental analysis (EA) of the CNGs was performed by using a Vario EL cube analyzer (Elementar, Hanau, Germany) for C, H, O, and N. The X-ray photoelectron spectroscopy (XPS) analysis of CNGs was performed using a ES-CALAB 250 spectrometer (VG Scientific, East Grinstead, UK) with Al Kα X-ray radiation as the X-ray source for excitation. The CNGs (100 μg mL^−1^) were fixed onto poly-D-lysine-coated slide to obtain fluorescence images under an optical microscope (Olympus BX61, Tokyo, Japan) with a digital camera (Olympus DP71). The average particle size of CNGs was determined using a transmission electron microscope (TEM; FEI Tecnai G2 F20 S-TWIN). 15 μL of CNGs (100 μg mL^−1^) was dropped onto a 300-mesh copper grid (formvar/carbon-coated) and dried under vacuum for 24 h. The lattice fringes of the CNGs were analyzed by DigitalMicrograph software (Gatan, Pleasanton, CA, USA). The cryo-electron microscope (cryo-EM) images of the CNGs prepared from an automated specimen-preparation device (FEI Vitrobot system) were recorded by Tecnai G2 F20 S-TWIN systems with cryo-holder (Philips/FEI, OG, USA). Briefly, the CNGs (1 mg mL^−1^; 10 μL) were dropped onto 300-mesh copper grid (formvar/carbon-coated) and held by tweezers in the Vitrobot system. The sample droplet was blotted with a filter paper, after which the sample was immediately plunged into liquid ethane to produce a vitrified sample, and then transferred to liquid nitrogen storage for 24 h before use. The CNGs on the copper grid were mounted onto SEM specimen stubs and sputtered with gold, then the SEM images were captured with a Hitachi S-4800 SEM (JEOL, Tokyo, Japan).

**Bacterial labeling**

Bacterial suspensions (10^7^ CFU mL^−1^) of *E. coli*, *S*. *aureus*, and *V. parahaemolyticus* were separately incubated with CNGs (100 μg mL^−1^) in 5 mM sodium phosphate (pH 7.4) at 25 ℃ under orbital shaking (150 rpm) for 1 h. The mixture was then centrifuged at 3,000 *g* for 5 min and washed three times with PBS solution or 5 mM sodium phosphate (pH 7.4) with 3% NaCl. Then, the CNGs-treated bacteria solution was dropped onto glass slides coated with poly-L-lysine (Thermo Fisher Scientific, Inc., Waltham, MA, USA) for fluorescence observation. The fluorescence images of untreated and treated bacteria under various excitation wavelengths (UV light; 330–385 nm), (blue; 450–480 nm), and (green light; 510–540 nm) were captured using an Olympus BX61 microscope (Tokyo, Japan) with an Olympus DP71 digital camera.

**TEM images of bacteria**

The suspensions (10^9^ CFU mL^−1^) of *E. coli*, *S*. *aureus*, and *V. parahaemolyticus* were centrifuged at 3,000 *g* for 5 min and washed three times with the respective buffer solution. Then, each bacterial culture was incubated with DAO/DEX_5.0_-CNGs (100 μg mL^−1^) for 1 h, and subsequently the mixtures were centrifuged at 3,000 *g* for 10 min and washed three times to remove the unbound DAO/DEX_5.0_-CNGs. The bacteria (10^4^ CFU mL^−1^) were fixed onto the copper grid and dried under vacuum for 1 h to observe using a TEM (HITACHI, Tokyo, Japan).

**Intracellular ROS assays**

The *V. parahaemolyticus* (10^7^ CFU mL^−1^) suspensions in sodium phosphate buffer (5 mM, pH 7.4) containing 3% NaCl were incubated with hydrogen peroxide (H_2_O_2_, 10 μg mL^−1^) and DAO/DEX_5.0_-CNGs (10−100 µg mL^−1^) at 25 ℃ for 3 h. Then the mixtures were centrifuged at 3,000 *g* for 10 min and washed three times with their respective buffer. Each of the bacterial suspension was further incubated with DCFH-DA (10 μM) at 25 ℃ for 30 min in dark, centrifuged (3,000 *g*, 10 min), and washed thrice to remove unbound DCFH-DA. 200 μL from each resuspended solution was transferred into a 96-well flat-bottom plate to measure the fluorescence intensity at excitation/emission wavelengths of 488/525 nm, respectively using a Synergy 4 multimode microplate spectrophotometer. The fluorescence images of *V. parahaemolyticus* using excitation (330−385 nm) and emission (420 nm) filters were acquired by an Olympus BX61 microscope with a digital camera (Olympus DP71).

***In vitro* cytotoxicity assays**

Cytotoxicity of DAO/DEX_5.0_-CNGs was investigated against four different human cell lines from lung fibroblast (IMR-90), embryonic kidney 293 (HEK-293T), liver epithelial (HepG2), and keratinocyte (HaCaT). Cell lines were obtained from American Type Culture Collection (ATCC, Manassas, VA, USA). HEK-293T & HaCaT and IMR-90 & HepG2 were cultured in Dulbecco’s modified Eagle’s medium (DMEM) and Eagle's Minimum Essential Medium (EMEM), respectively, and were supplemented with fetal bovine serum (FBS, 10%), antibiotic-antimycotic (1%), L-glutamine (2 mM), and nonessential amino acids (NEAA, 1%), and incubated with 5% CO_2_ at 37 ℃. The cell number was determined by the trypan blue exclusion method. The cell viability was determined using an alamarBlue assay. Briefly, each of the cell lines (*ca.* 1.0 × 10^4^ cells per well) were separately seeded in 96-well plates with respective culture medium containing 5% CO_2_ at 37 ℃ for 12 h. Then, 100 μL of the culture medium from each well was replaced with a fresh medium containing various concentrations of DAO/DEX_5.0_-CNGs (0−100 μg mL^−1^) and further incubated with 5% CO_2_ at 37 ℃ for 48 h. The cells were carefully rinsed thrice with sterile PBS solution and treated with the alamarBlue reagent (ten-fold dilution, 100 μL per well) for 4 h at 37 ℃. The fluorescence intensities at excitation/emission wavelength of 560/590 nm, respectively were measured using Synergy 4 multimode microplate spectrophotometer.

**Hemolysis assays**

The blood sample was collected from a healthy volunteer (male, 25 years old) in a sterile test tube containing ethylenediaminetetraacetic acid (EDTA). The red blood cells (RBCs) collection procedure was in accordance with institutional guidelines and relevant laws. The RBCs were centrifuged (3,000 *g*, 10 min, 4 ℃) immediately and washed thrice (PBS; pH 7.4) to remove the serum. The DAO/DEX_5.0_-CNGs (1−100 µg mL^−1^) were incubated with RBCs (*ca.* 4.0 vol% blood cells) at 37 ℃ for 1 h. Then, the aliquots were centrifuged at 1,000 *g* for 10 min and absorbance at 576 nm was recorded from the supernatant. RBCs treated with PBS (pH 7.4) and DI water were used as a negative and positive control, respectively. The hemolysis (%) was calculated using the following equation:

Hemolysis (%) = [(OD_576_ _DAO/DEX5.0-CNGs_ − OD_576_ _blank_)/(OD_576_ _deionized water_ − OD_576_ _blank_)]×100%

**Histopathological analysis of shrimp hepatopancreas**

Histological analysis was performed through staining hepatopancreas of shrimp with hematoxylin and eosin stain (H&E). After *V. parahaemolyticus* challenge assay (Day 3), the shrimp were euthanized and the hepatopancreas was harvested from the untreated and treated groups. Then, the sections embedded in the optimal cutting temperature compound (Sakura Finetek, Torrance, CA, USA) were cryo-sectioned at 6 µm thickness onto Superfrost Plus Glass Slides (Thermo Fisher Scientific, Inc., Waltham, MA, USA) (*n* = 5). The hepatopancreas from all the groups was stained with H&E and fixed with multi-medium to examine under an Olympus 1X71 microscope (Tokyo, Japan).

**Shrimp hemocytes RNA extraction and cDNA synthesis**

After the *V. parahaemolyticus* challenge assay, each group of shrimps was euthanized on day 1. The shrimp hemocytes were collected and the RNA extraction protocol was followed as per the manufacturer’s instruction for the Direct-zol RNA Miniprep Kits (Zymo Research, Irvine, CA, USA). The shrimp blood samples were centrifuged at 3000 *g* for 10 min to remove the supernatant, to which 400 μL of Trizol (95% v/v ethanol) was added to dissolve the pellets. The aliquots were subsequently loaded into the extraction column. After washing, In-Column DNase I digestion, and RNA elution by diethyl pyrocarbonate (DEPC) water was performed. The extracted RNA was stored at −80 ℃ when not in use. The extracted RNA (1 μg) was used to synthesize cDNA according to the manufacturer’s instruction for the RevertAid RT Reverse Transcription Kit (Thermo Fisher Scientific, Inc., Waltham, MA, USA).

**Real-time PCR analysis**

All primers used in this study are shown in **Table S4**. The FastStart Universal SYBR Green Master (ROX) reagents were quantified for gene expression by an Agilent Technologies (Santa Clara, CA, USA) Stratagene Mx3000P for real-time PCR. The thermal profile was as follows: pre-incubation at 95 ℃ for 10 min, then the amplification process, followed by the denaturing step at 95 ℃ for 30 s, annealing step at 60 ℃ for 20 s, and extension step at 72 ℃ for 1 min for 40 cycles. Analysis of the melting curve confirmed the purified product after PCR.

**Microbiota analysis**

After the *V. parahaemolyticus* challenge assay, each group of shrimp was euthanized on day 1. We collected the stool sample from the midgut of white-leg shrimp and stored it at −80 ℃ for subsequent microbiota analysis. The DNA extraction and microbiota analysis from the stool samples were conducted by the Biotools Biotechnology Company (Taipei, Taiwan). The effective tags were obtained from the sequence data processing by FLASH, QIIME, UCHIME Algorithm, and USEARCH software [2−6], and the UPARSE software was used to get the sequence assignment in the data set with ≧ 97% similarity to the same operational taxonomic units (OTUs) [7,8]. The DNA sequence with species annotation was used for RDP 3 classifier software and Silva Database v.132 [9,10]. The OTUs abundance information was normalized using QIIME. Subsequent analysis of alpha and beta diversity, relative abundance, and evenness accounting for diversity was conducted. These data were also used for the top 100 OTU heat map and taxonomic composition.

**Expression and purification of toxin recombinant protein**

The *V. parahaemolytic*us pirA and pirB toxin proteins were obtained by molecular cloning and recombinant protein expression techniques. Briefly, the genes are amplified by PCR and cloned into pET21a plasmids with the restriction enzyme *Eco*RI and *Xho*I using Gibson assembly kit (NEB; Ipswich, MA, USA). The plasmids were transformed into Rosetta (DE3) competent cells of *E. coli* to express recombinant pirA (rPirA) and recombinant pirB (rPirB) protein. The recombinant protein was induced by treating with isopropyl thiogalactoside (IPTG, 4 x 10^−1^ μM) at 16 ℃ for 16 h under orbital shaking (150 rpm). The recombinant protein of rPirA and rPirB with 6x His-tag were purified using nickel NTA agarose resin (ABT, FL, USA). Purification conditions were as per the user manual, and the purified rPirA and rPirB were concentrated with Amicon^®^ ultra-15 centrifugal filters (Merck Millipore, Burlington, MA, USA), and dialyzed in PBS solution. The protein concentration was measured using Pierce BCA Protein Assay Kit (Thermo Fisher Scientific, Waltham, MA, USA).

**The toxin adsorption assay of CNGs**

As-prepared DAO/DEX_5.0_-CNGs (0.5−10 mg mL^−1^) were mixed with 0.1 mg of rPirA or rPirB in PBS solution (pH 7.4) at 25 ℃ for 30 min. Then, the mixtures were centrifuged at 10,000 *g* to remove the absorbed toxins. The supernatant (20 μL) was electrophoresed in 12% SDS-PAGE gel, and then the separated proteins were transferred to polyvinylidene difluoride (PVDF) membrane (400 mA, 1.5 h). The membrane was blocked with blocking buffer [3% (wt./vol.) skim milk in 0.5% TBST buffer (0.5 M NaCl, 50 mM Tris·HCl, pH 7.5, 0.5% Tween 20)] for 1 h at room temperature and then incubated with His-tag antibody (1:5000; GeneTex Inc., Irvine, CA, USA) for 1 h at room temperature. The membrane was then washed three times with 0.5% TBST buffer and incubated with corresponding horseradish peroxidase (HRP)-conjugated sheep anti-mouse antibody (1:5000; GeneTex) for 1 h at room temperature. The membranes were washed again four times with 0.5% TBST and detected by ECL Western Blotting Detection Reagents (GE Healthcare Bio-Sciences, Piscataway, NJ, USA).

***In vivo* fluorscence imaging of shrimp organs for bioaccumulation studies**

The DAO/DEX_5.0_-CNGs (0.1 mg mL^−1^) and rhodamine B isothiocyanate (RITC, 0.01 mg mL^−1^) were mixed in 5.0 mM sodium phosphate buffer (pH 9). After stirring for 24 h, the mixture was centrifuged at a RCF of 35,000 *g* for 1 h to remove free RITC. After three centrifuge/wash cycles, the colloids were resuspended separately in 5.0 mM sodium phosphate buffer (pH 7.4) and stored in refrigerator (4 ° C). The fluorescence spectra of RITC-DAO/DEX_5.0_-CNGs was recorded using a monochromatic microplate spectrophotometer (Synergy 4 Multi-Mode; Biotek Instruments, Winooski, VT, USA) under excitation at 530 nm and the quantum yield was calculated as per the below equation.

$$\psi=\Psi_{R}\times\frac{I}{I_{R}}\times\frac{A_{R}}{A}\times\frac{n^{2}}{{n_{R}}^{2}}$$

where $\psi$ and $\Psi_{R}$ are the quantum yield of RITC-DAO/DEX_5.0_-CNGs and standard (RITC), respectively. *I* represents fluorescence intensity, and $A$ is the absorbance at the excitation wavelength. $n$ indicates the refractive index of the solvent.

Subsequently, the DAO/DEX_5.0_-CNGs-RITC (100 μg g^−1^) was mixed with a commercial feed and fed the shrimp for 7 days. To analyze the bioaccumulation of DAO/DEX_5.0_-CNGs in shrimp, they were placed in an IVIS Lumina II system (Caliper Life Sciences, Inc., Massachusetts, USA), and and dorsal and organ images were acquired for quantifying signal intensity.

**Statistics**

Results were expressed as mean ± standard deviation. Comparative studies of means were carried out using a one-way analysis of variance (one-way ANOVA). Signiﬁcance was accepted with *p* < 0.05.

**Additional Figures**


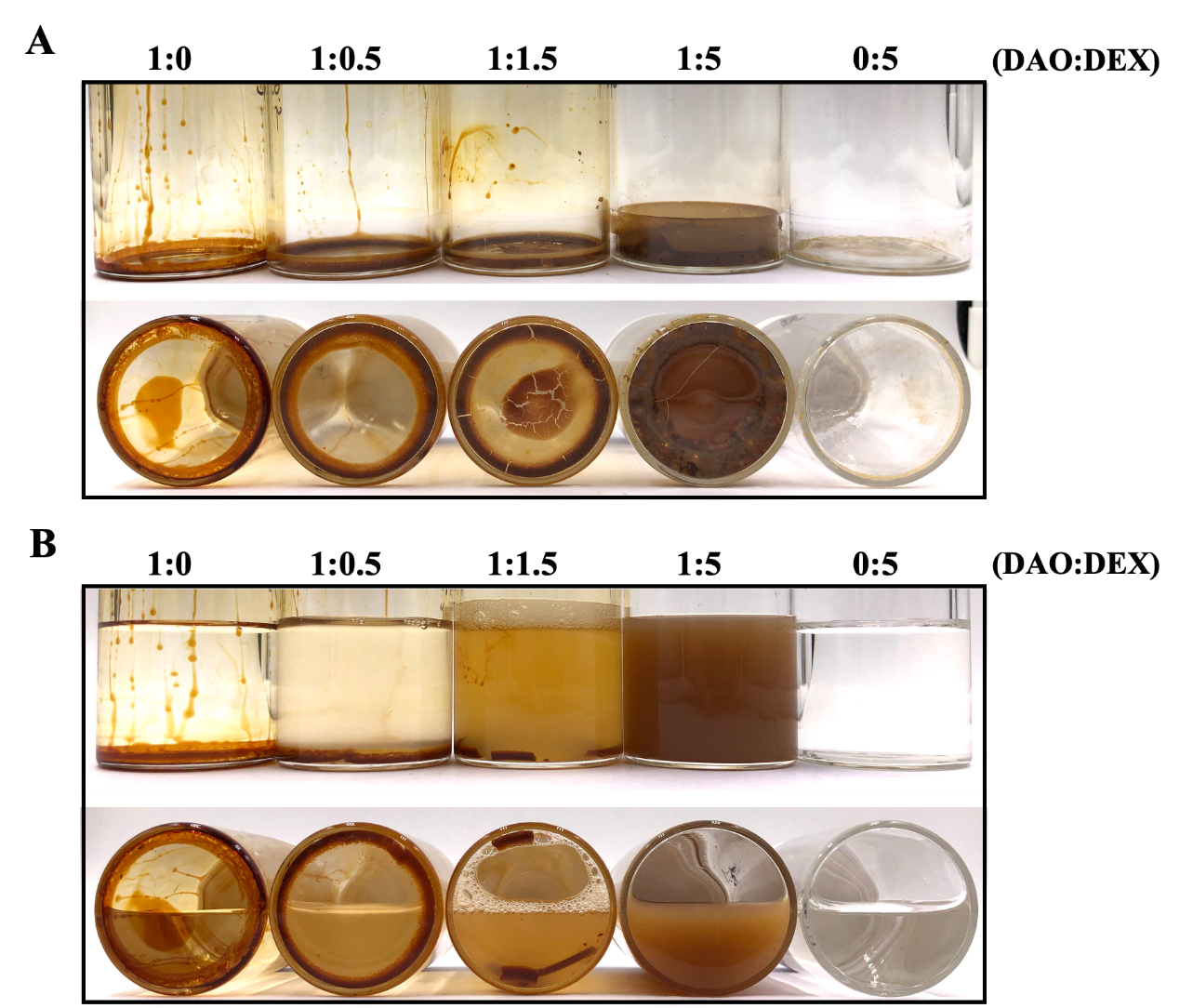


**Fig. S1** The photographs of (A) as-prepared products and (B) after being dissolved in deionized water (4 mL) for mixtures of 1,8-diaminooctane (DAO) and dextran 70 (DEX) heated at 180 ℃ for 3 h.


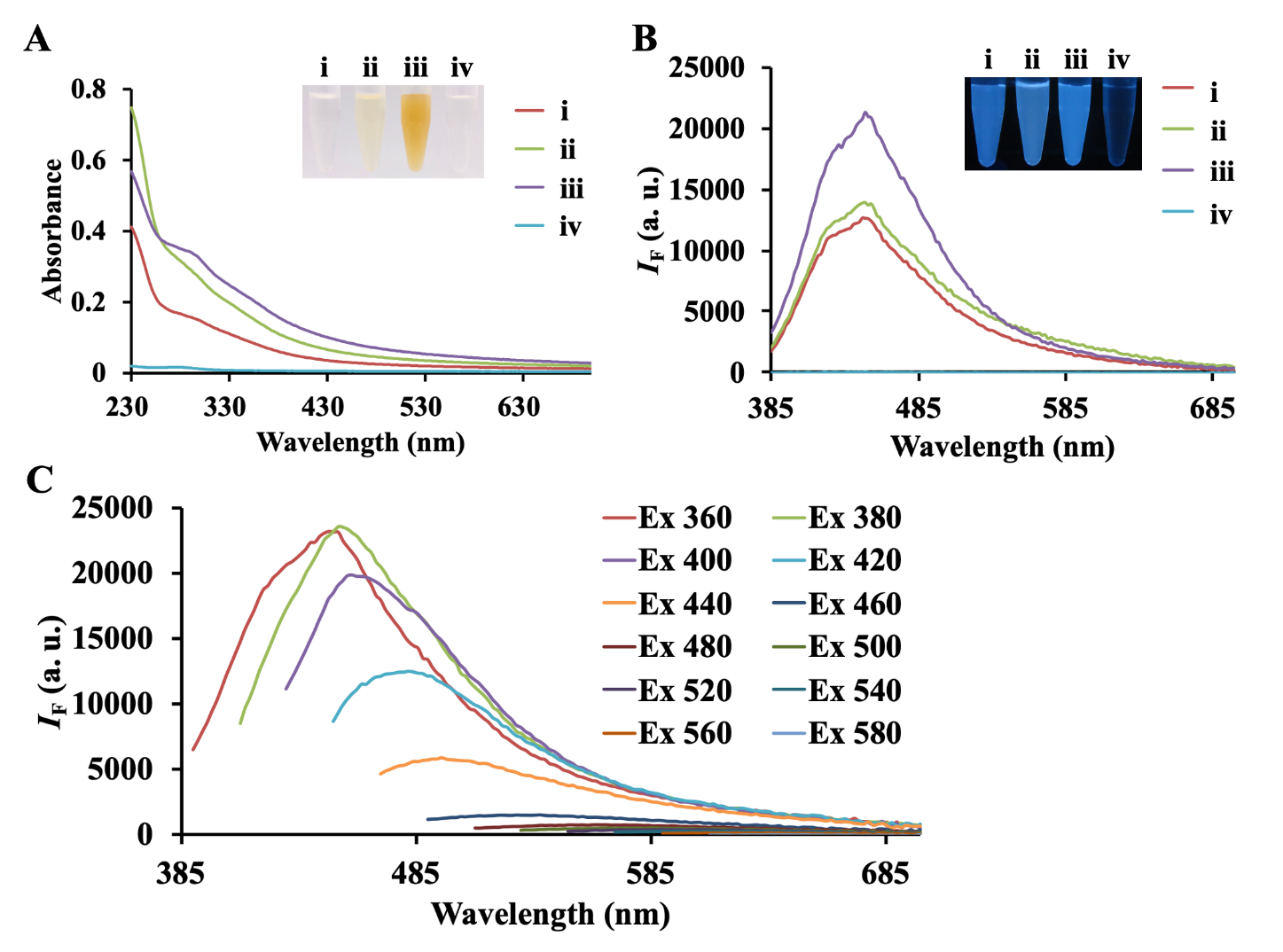


**Fig. S2** (A) UV-Vis absorption and (B) fluorescence spectra of i DAO/DEX_0.5_-CNGs, ii DAO/DEX_1.5_-CNGs, iii DAO/DEX_5.0_-CNGs, and iv product obtained by heating dextran with a concentration of 0.5 mg mL^−1^ in 5 mM sodium phosphate buffer (pH 7.4). (**C**) Excitation-dependent fluorescence spectra of DAO/DEX_5.0_-CNGs (0.5 mg mL^−1^) in 5 mM sodium phosphate buffer (pH 7.4). The excitation wavelength set for the fluorescence measurement (B) was 365 nm. Inset to (A) and (B): photographs of the corresponding solutions (A) before and (B) after excitation under a hand-held UV lamp (365 nm). Fluorescence intensity (*I*_F_) is plotted in arbitrary units (a. u.).


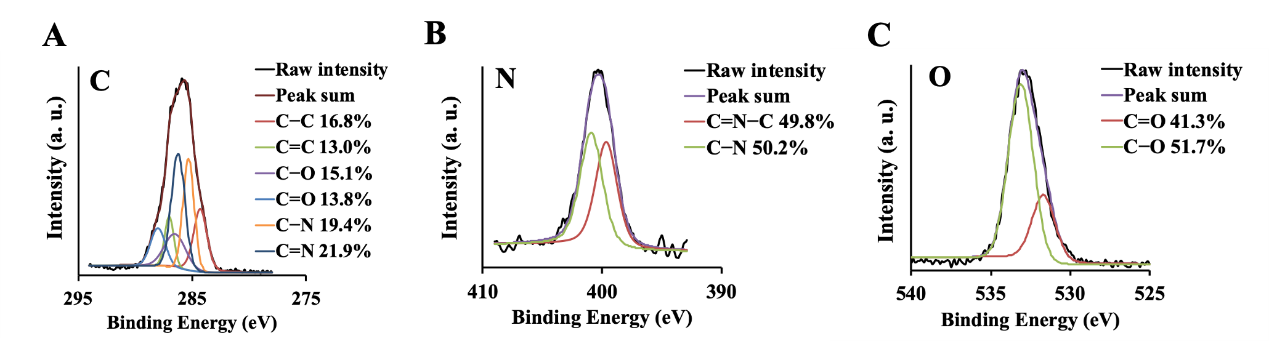


**Fig. S3** The XPS spectra of (A) C1s, (B) N1s, and (C) O1s of the purified DAO/DEX_5.0_-CNGs.


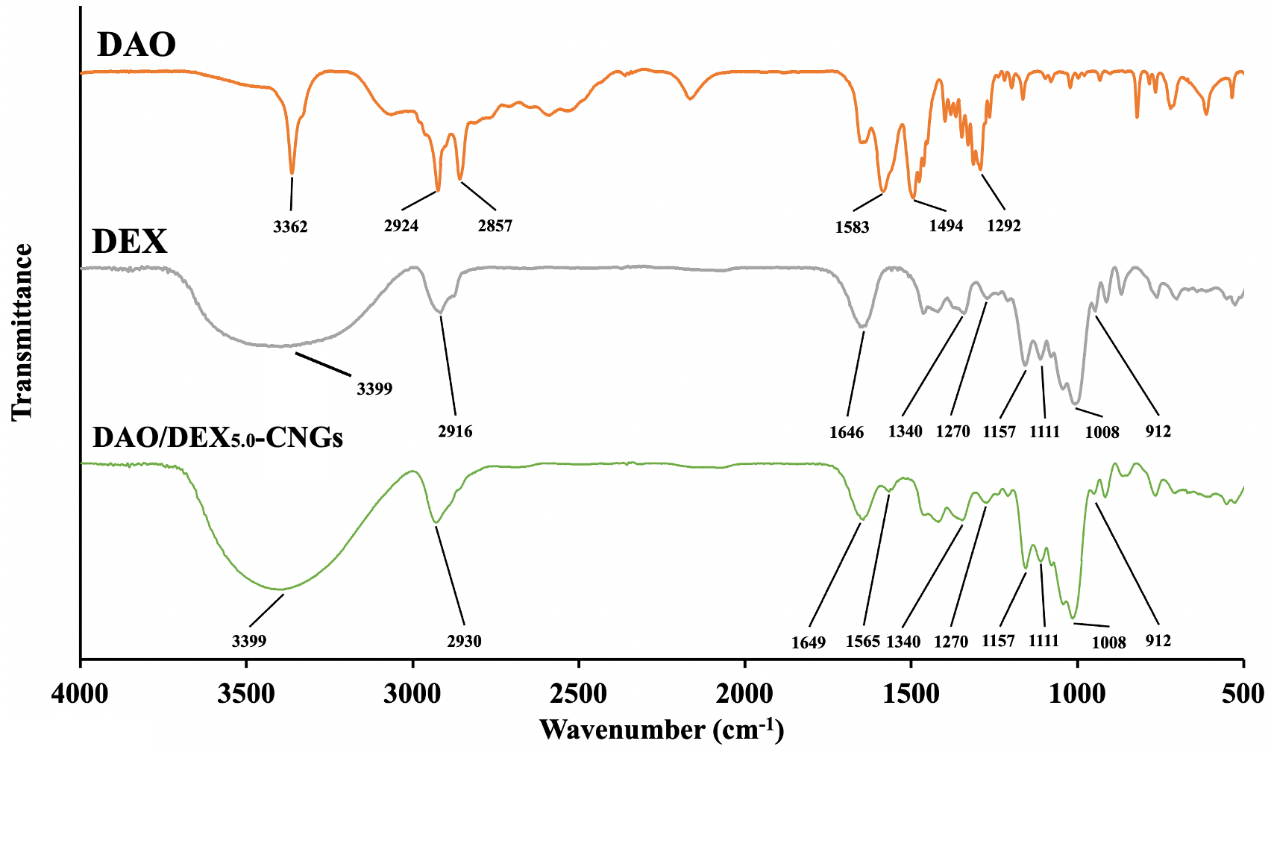


**Fig. S4** The FT-IR spectra of the 1,8-diaminooctane (DAO), dextran 70 (DEX), and DAO/DEX_5.0_-CNGs.


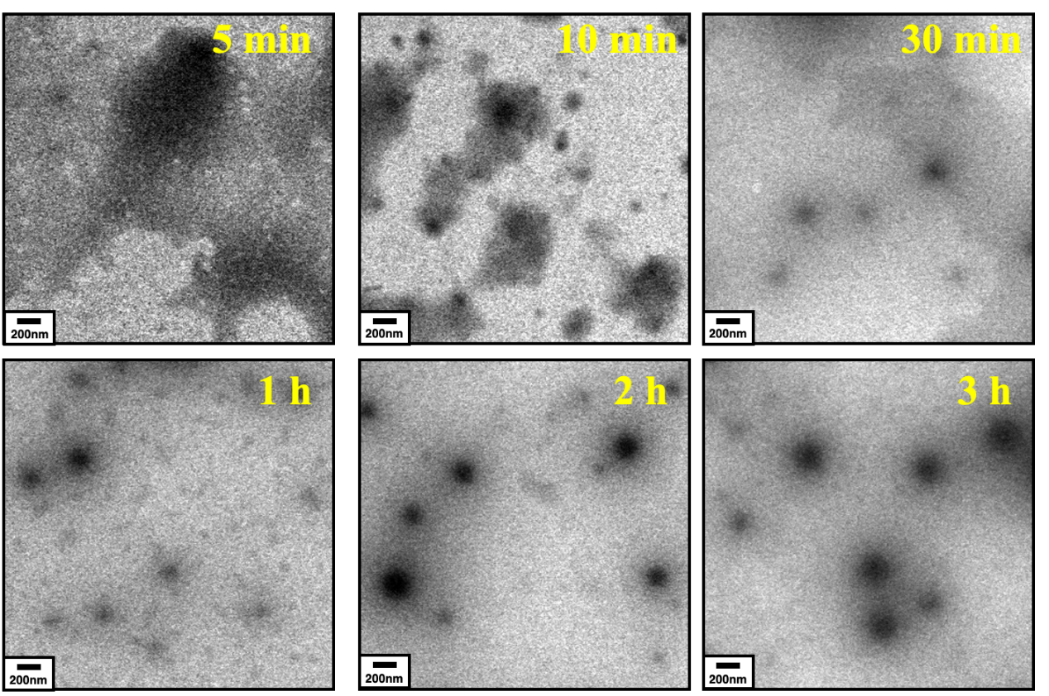


**Fig. S5** TEM images of the DAO/DEX_5.0_-CNGs were obtained by heating a mixture of DAO and DEX at 180 ℃ for 5 min, 10 min, 30 min, 1 h, 2 h, and 3 h.

***
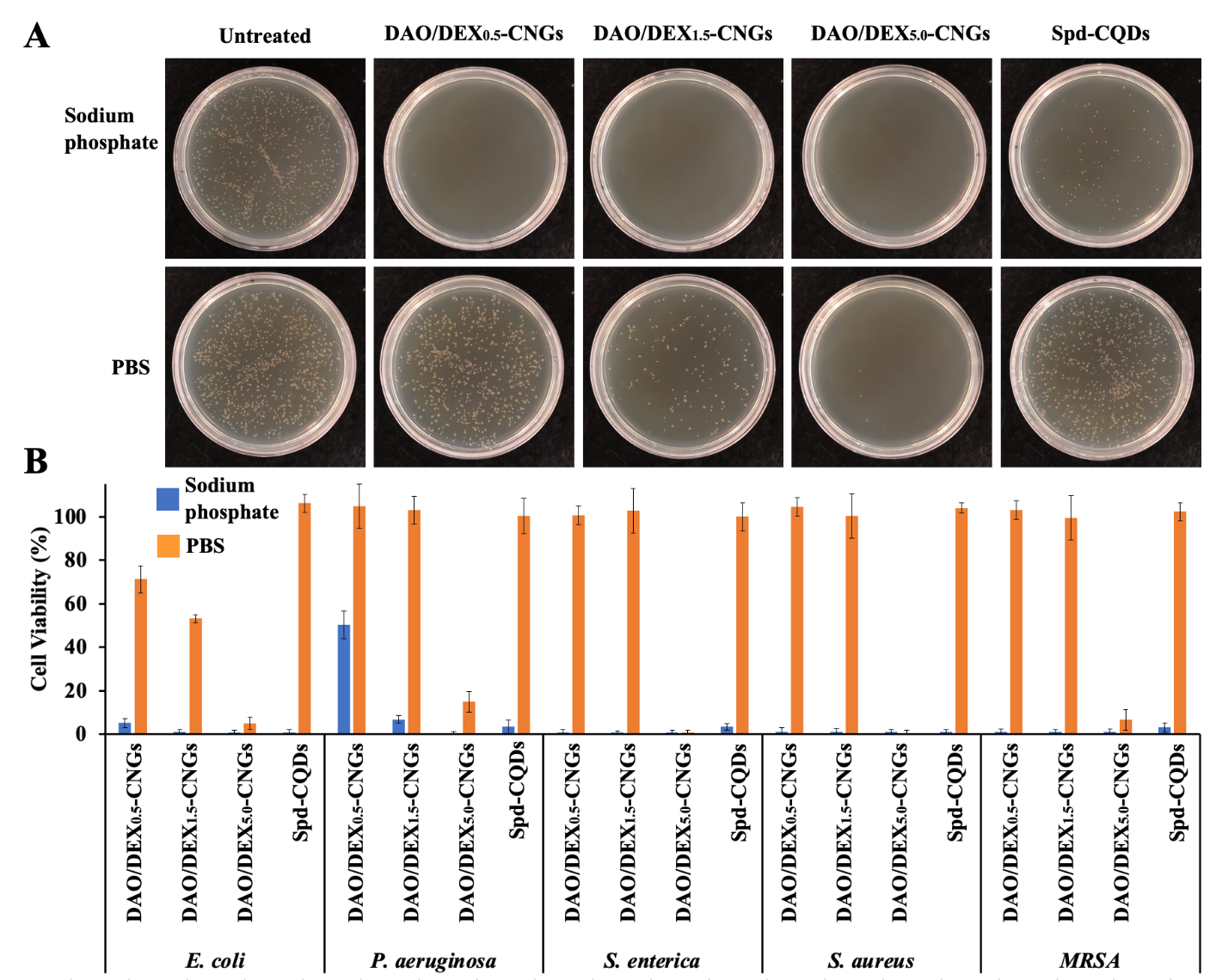
***

**Fig. S6** (A) Representative colony formation of *E. coli* on Luria-Bertani (LB) agar plates untreated or treated with DAO/DEX-CNGs or Spd-CQDs at a concentration of 10 μg mL^−1^ in 5 mM sodium phosphate buffer (pH 7.4) or PBS (137 mM NaCl, 2.7 mM KCl, 10 mM Na_2_HPO_4_, and 2.0 mM KH_2_PO_4_; pH 7.4) solution. (B) Relative cell viability of bacteria after being treated with DAO/DEX-CNGs or Spd-CQDs (10 μg mL^−1^) in 5 mM sodium phosphate buffer (pH 7.4) or PBS solution. Error bars are the standard deviations of three repeated experiments.


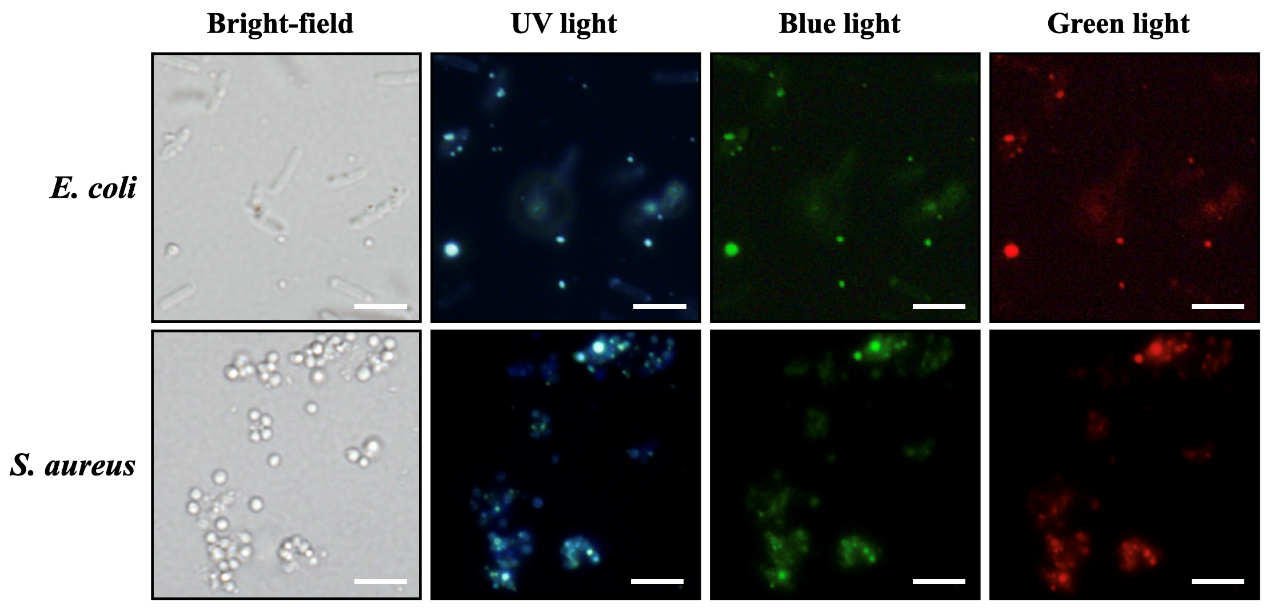


**Fig. S7** Bright-field and fluorescent images of *E. coli* (10^7^ CFU mL^−1^) and *S. aureus* (10^7^ CFU mL^−1^) under excitation wavelengths of UV light (360–380 nm), blue light (460–480 nm), and green light (510–530 nm) after incubation with the DAO/DEX_5.0_-CNGs (100 μg mL^−1^) in PBS for 1 min.

***
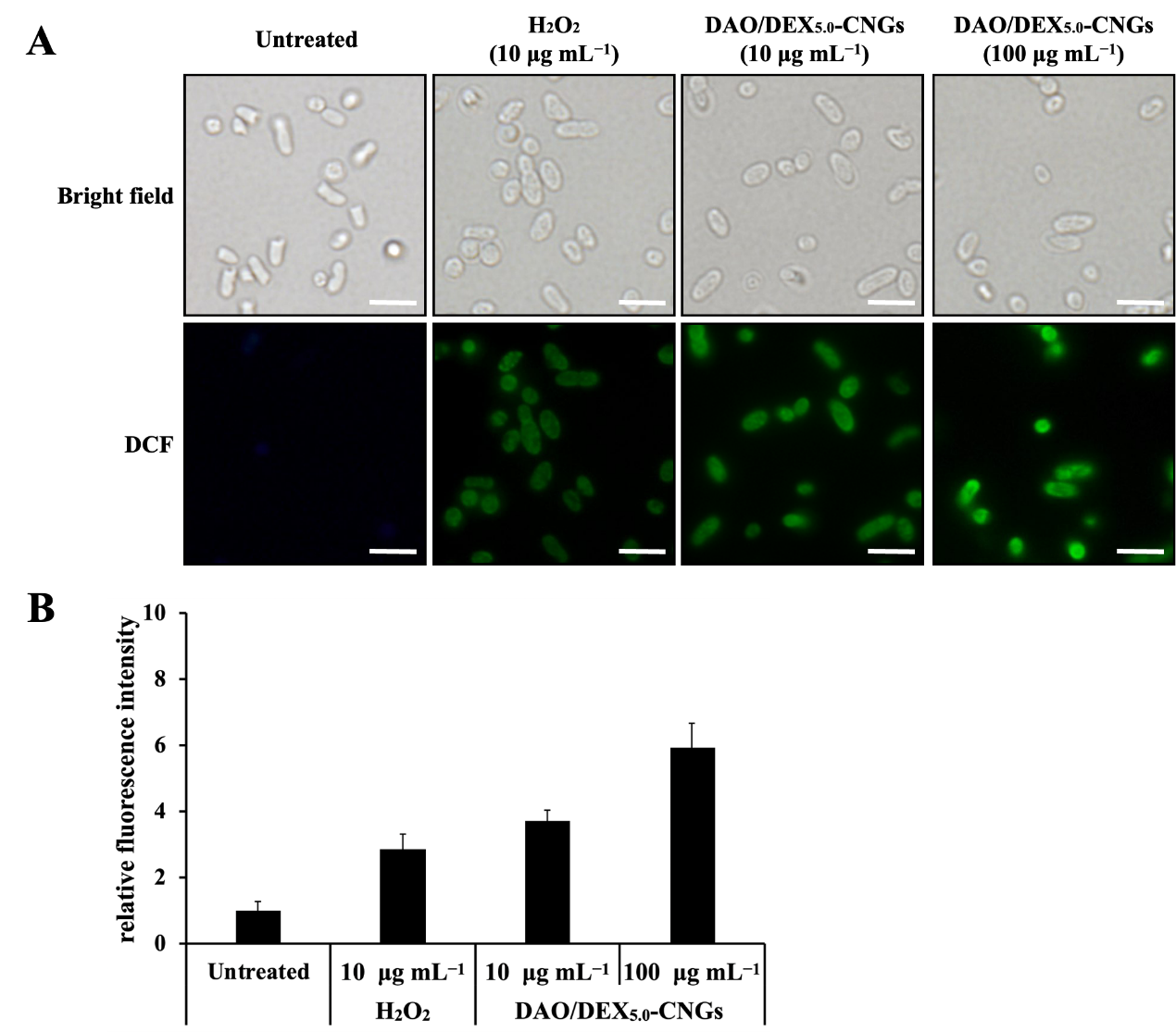
***

**Fig. S8** (A) Bright-field and fluorescent images of *V. parahaemolyticus* cells (1.0 × 10^7^ CFU mL^−1^) after staining with DCFH-DA to assess the level of ROS without (untreated) and after treatment with H_2_O_2_ (10 μg mL^−1^), DAO/DEX_5.0_-CNGs (10 μg mL^−1^) or DAO/DEX_5.0_-CNGs (100 μg mL^−1^) for 60 min. *V. parahaemolyticus* was treated with H_2_O_2_ (10 μg mL^−1^) as a positive control. (B) Quantification of cellular ROS level using DCF fluorescent intensity. Error bars are the standard deviations of three repeated experiments.

***
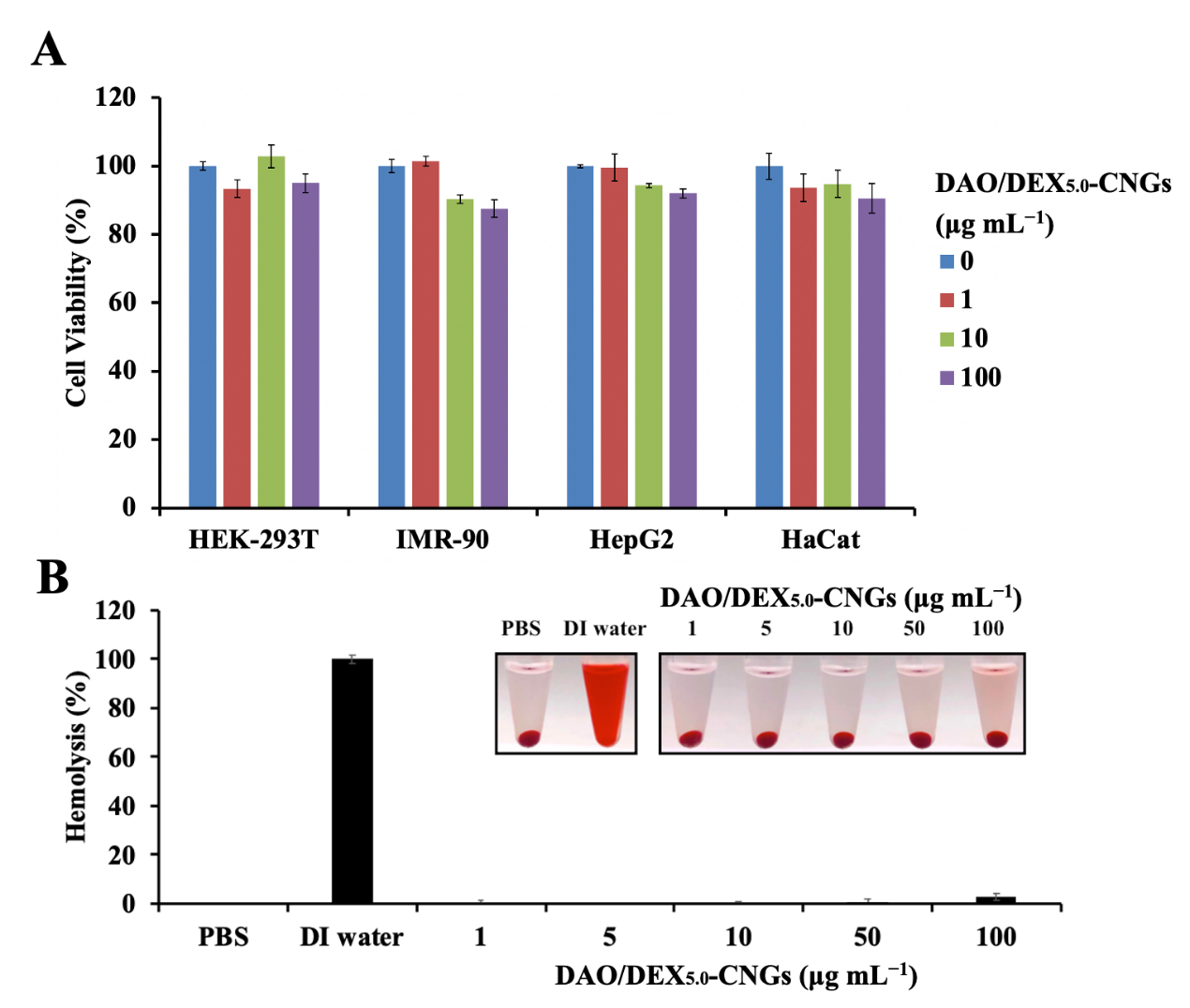
***

**Fig. S9** (A) Relative cell viability of HEK-293T, IMR-90, HepG2, and HaCat cells after incubation with various concentrations of DAO/DEX_5.0_-CNGs for 48 h at 37 ℃. (B) Hemolytic activities of DAO/DEX_5.0_-CNGs against RBCs dispersed in PBS. The solutions containing RBCs with PBS and DI water were used as a negative and positive control experiment, respectively. Insets: photographs of RBC solutions after treatment with PBS, DI water, or DAO/DEX_5.0_-CNGs. The error bars represent the standard deviation of three repeated measurements.


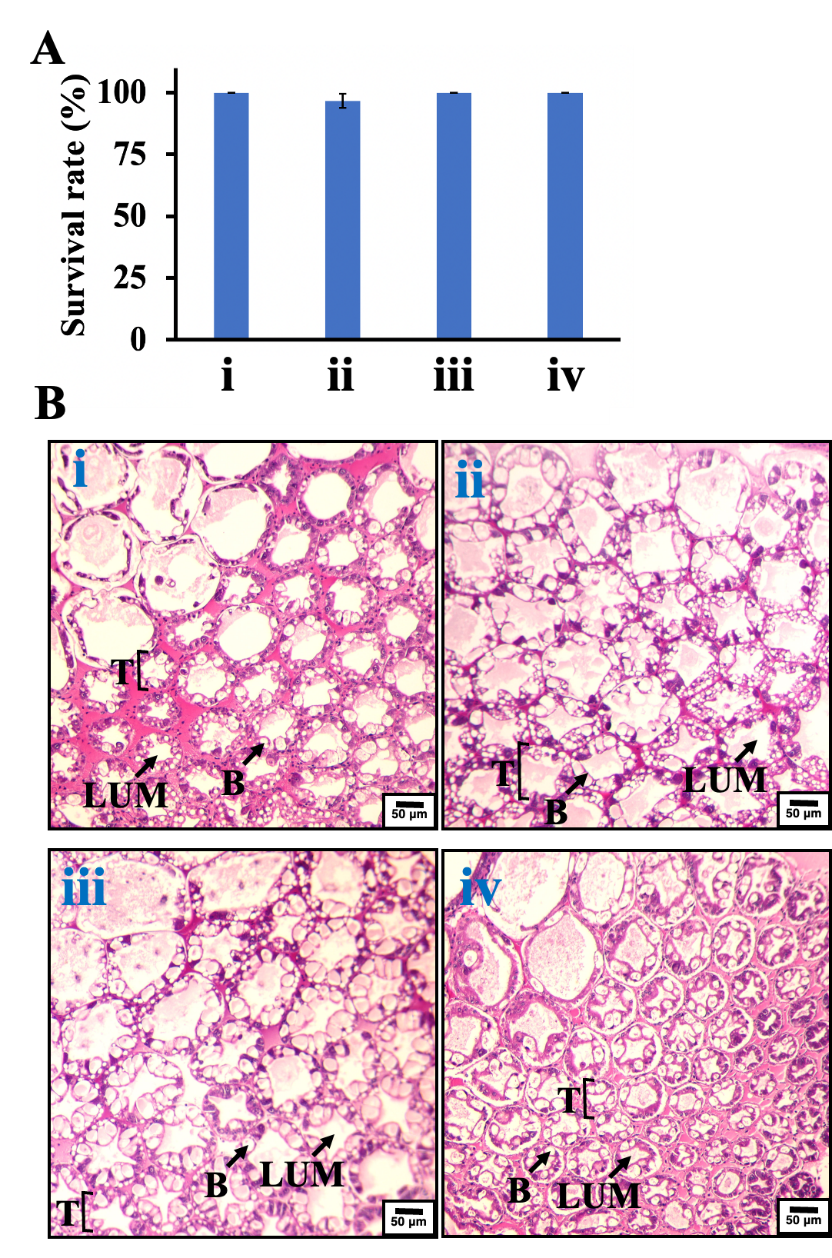


**Fig. S10** (A) Survival rate of post-larval shrimp when continuously fed with (i) a commercial feed or (ii to iv) commercial feed with additive DAO/DEX_5.0_-CNGs of (ii) 1.0 μg g^−1^, (iii) 10 μg g^−1^ and (iv) 100 μg g^−1^ for 7 days. (B) Cross-sectional micrograph of H&E stained hepatopancreas of the shrimp fed with commercial feed without additive (i) or with DAO/DEX_5.0_-CNGs additives of 1.0 (ii), 10 (iii) and 100 (iv) μg g^−1^ at day 7. Tubule (T); Lumen (Lum); Blasenzellen cells (B-cells, B). The error bars in (A) represent the standard deviation of three repeated measurements.


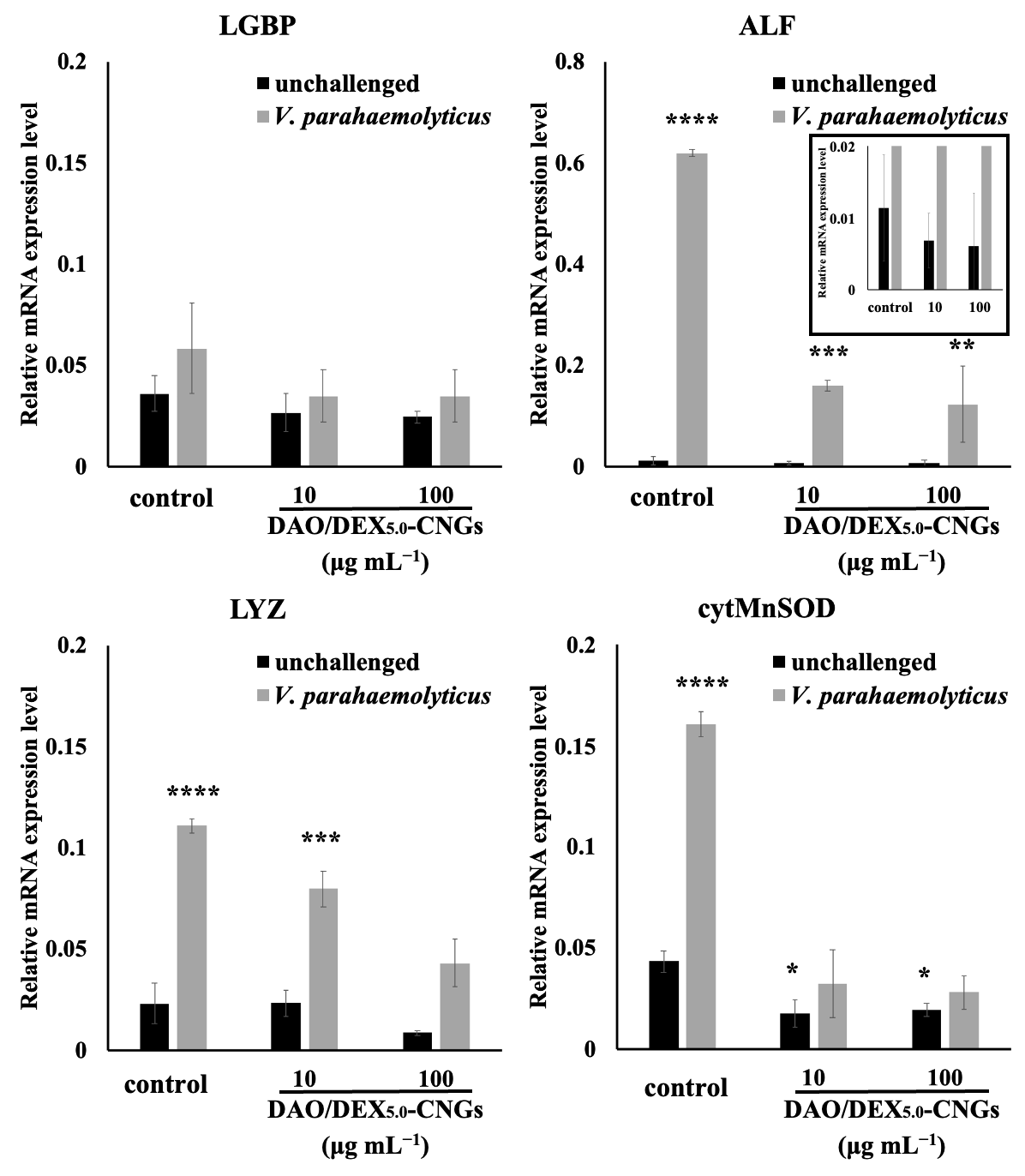


**Fig. S11** Gene expression levels of LGBP, ALF, LYZ, and cytMnSOD in the hemocytes from the *V. parahaemolyticus*-infected shrimp after 24 h. The shrimp have been fed with different doses of DAO/DEX_5.0_-CNGs for 3 days before challenging with *V. parahaemolyticus*. An asterisk indicates statistically significant differences (**p* < 0.05, ***p* < 0.01, ****p* < 0.001, *****p* < 0.0001; *n* = 3) as compared with untreated group (without fed DAO/DEX_5.0_-CNGs and without *V. parahaemolyticus* challenge). The inset to ALF shows the gene expression level of uninfected shrimp.


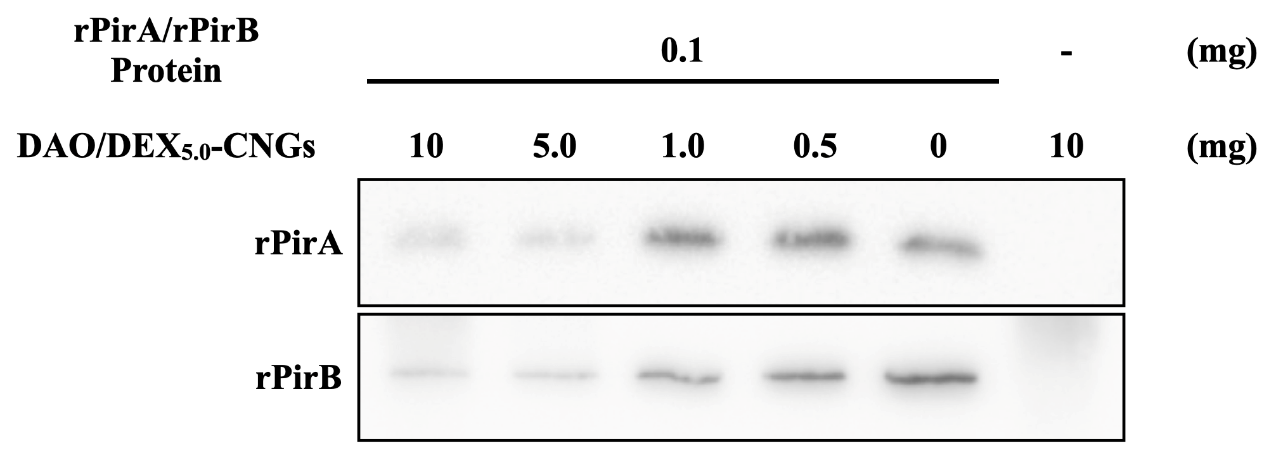


**Fig. S12** The toxin adsorption assay showed that the recombinant toxin proteins, PirA (rPirA; 0.1 mg) or PirB (rPirB; 0.1 mg), were adsorbed by DAO/DEX_5.0_-CNGs (0.5−10 mg).

***
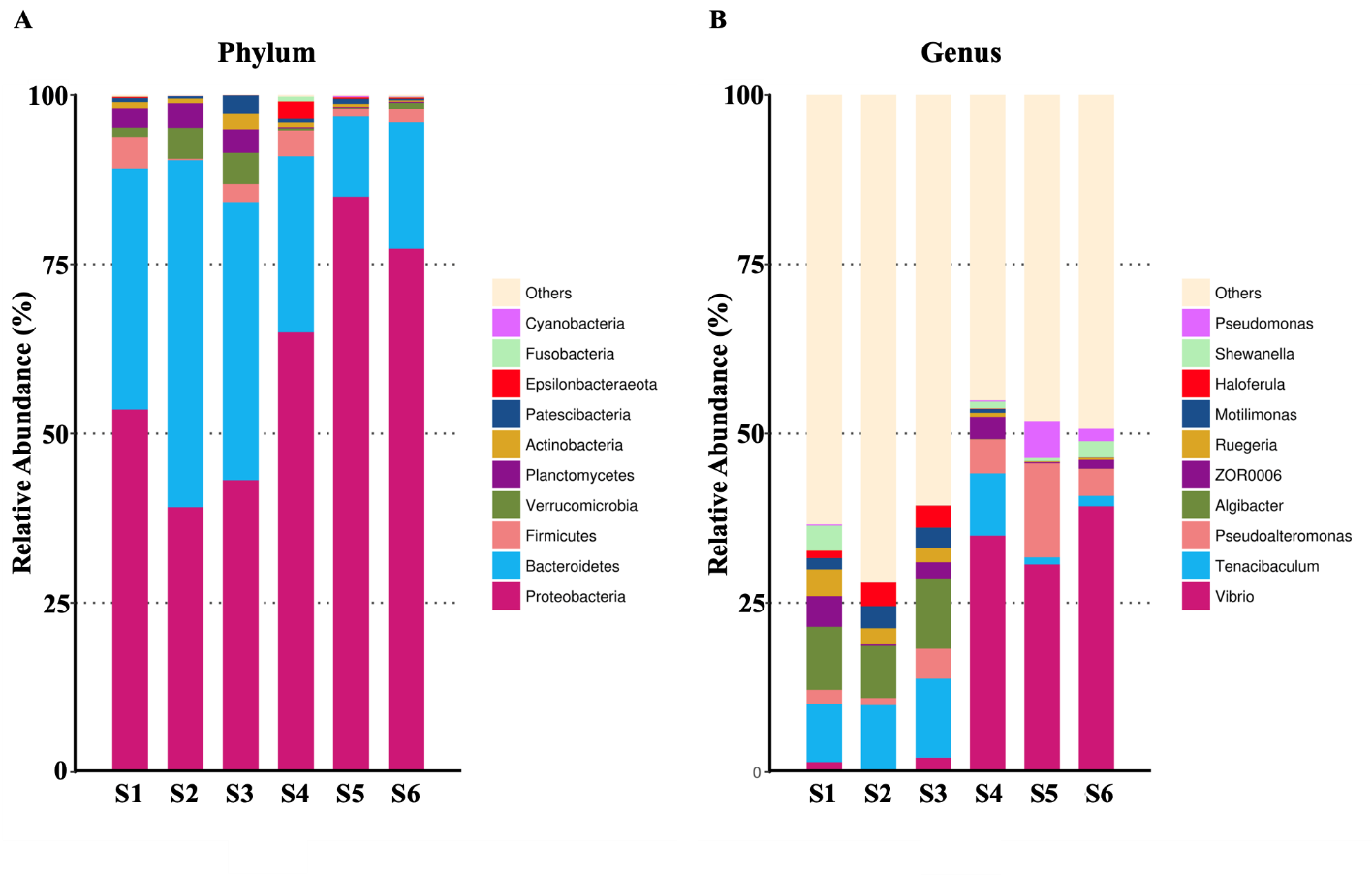
***

**Fig. S13** Microbiota composition at the (A) phylum and (B) genus level with relative abundance for the top ten. (S1 and S4) commercial feed, and commercial feed with DAO/DEX_5.0_-CNGs additives of (S2 and S5) 10 μg g^-1^ or (S3 and S6) 100 μg g^-1^; (S4 to S6) *V. parahaemolyticus* challenge after a feed.


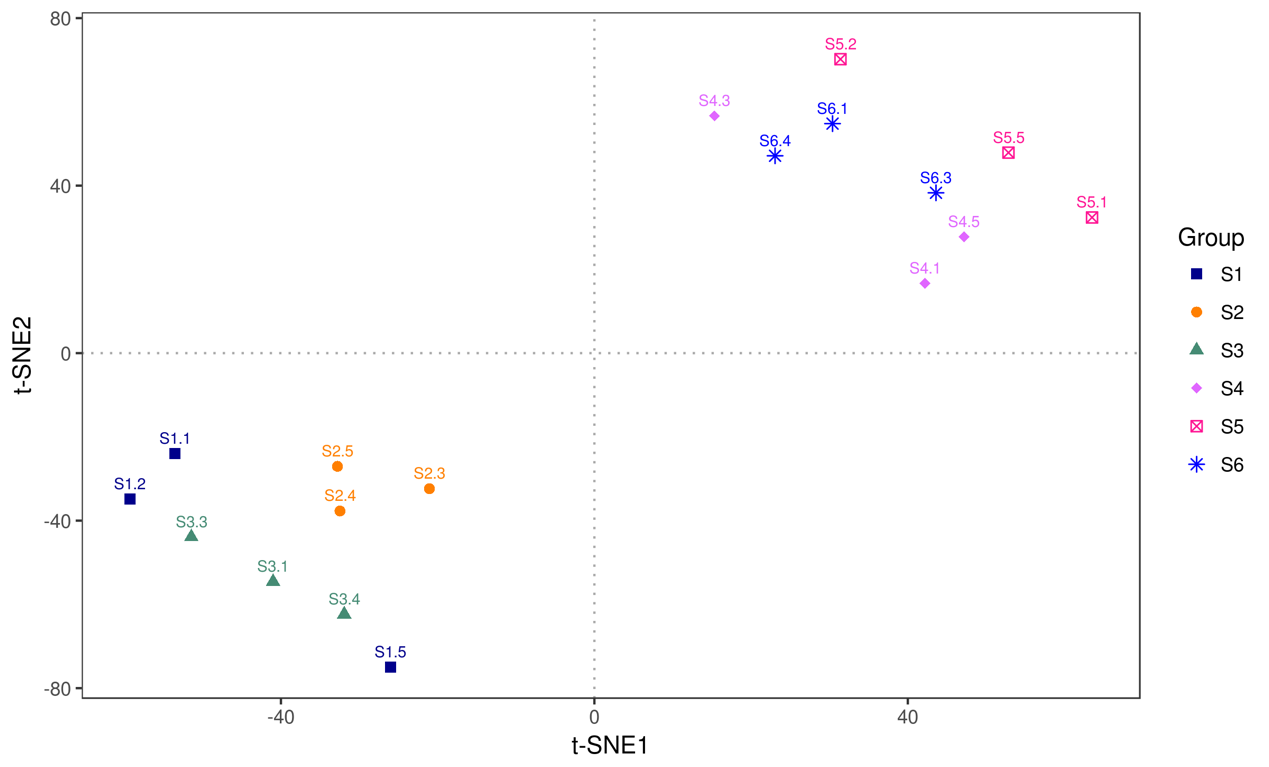


**Fig. S14** The t-SNE analysis shows the similarity of each sample. (S1 and S4) commercial feed, and commercial feed with DAO/DEX_5.0_-CNGs additives of (S2 and S5) 10 μg g^-1^ or (S3 and S6) 100 μg g^-1^; (S4 to S6) *V. parahaemolyticus* challenge after a feed.


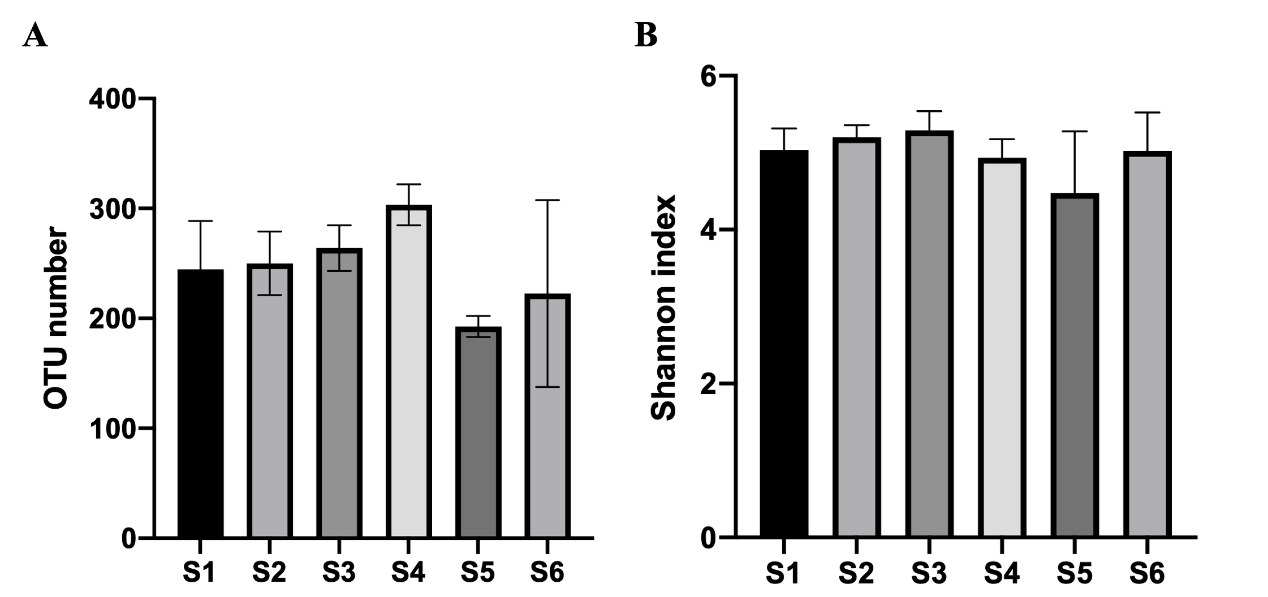


**Fig. S15** (A) OTU number and (B) microbial diversity of gut microbiota from whiteleg shrimp fed with commercial feed in the absence or presence of DAO/DEX_5.0_-CNGs additives without or with *V. parahaemolyticus* infection. (S1) commercial feed, and commercial feed with DAO/DEX_5.0_-CNGs additives of (S2) 10 μg g^-1^ or (S3) 100 μg g^-1^; (S4) commercial feed followed by *V. parahaemolyticus* challenge, and commercial feed with DAO/DEX_5.0_-CNGs additives of (S5) 10 μg g^-1^ or (S6) 100 μg g^-1^ followed by *V. parahaemolyticus* challenge.


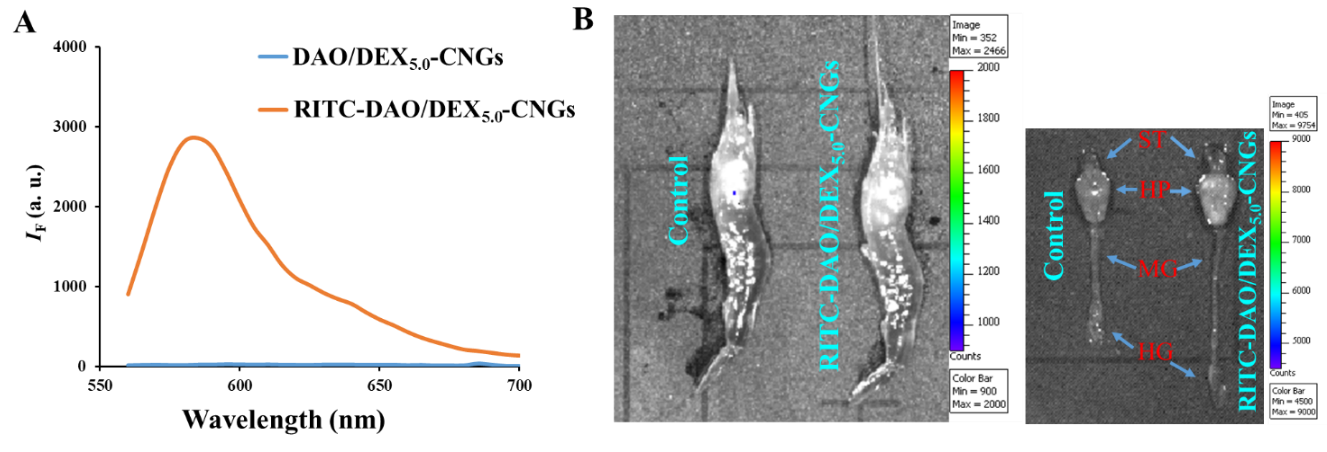


**Fig. S16** The bioaccumulation of DAO/DEX5.0-CNGs in shrimp. (A) The fluorescent spectra of DAO/DEX5.0-CNGs and RITC-labelled DAO/DEX5.0-CNGs with an excitation at 530 nm. (B) The fluorescent images of shrimp organs on IVIS platform after feeding RITC-labelled DAO/DEX5.0-CNGs-RITC (100 μg g−1) for 7 days. ST: stomatch, HP: hepatopancreas, MG: midgut, HG: hindgut.

**Additional Tables**

**Tab. S1** Product yields, fluorescence quantum yields, hydrodynamic diameters, zeta potentials, and elemental compositions of the as-prepared products from DAO, DEX or the mixture of DAO and DEX with mass ratios (DAO:DEX) of 1.0:0.5, 1.0:1.5, and 1.0:5.0 prepared by dry heating at 180 ^o^C for 3 h.

| **DAO/DEX** | **Yield (%)** | **Quantum Yield**  **(%)** | **Hydrodynamic Diameter**  **(nm)** | **Zeta Potential (mV)** | **Elemental Composition (%)** | | | |
| --- | --- | --- | --- | --- | --- | --- | --- | --- |
|  |  |  |  |  | **C** | **N** | **O** | **H** |
| **DAO** | < 0.1 | - | - | - | - | - | - | - |
| **DAO:DEX (1.0:0.5)** | 15.1 | 0.51 | 208.9 ± 14.4 | 13.2 ± 1.3 | 40.6 | 1.9 | 50.0 | 7.5 |
| **DAO:DEX (1.0:1.5)** | 39.5 | 0.57 | 321.5 ± 9.4 | 6.1 ± 1.5 | 39.2 | 1.2 | 52.3 | 7.2 |
| **DAO:DEX (1.0:5.0)** | 70.7 | 0.88 | 580.2 ± 13.8 | 5.8 ± 0.5 | 38.3 | 0.8 | 53.8 | 7.2 |
| **DEX** | 73.7 | < 0.01 | - | −5.5 ± 3.4 | 36.8 | 0.1 | 56.2 | 6.9 |

**Tab. S2** Selected band assignment for FT-IR spectra of 1,8-diaminooctane, dextran, and DAO/DEX_5.0_-CNGs.

| **1,8-Diaminooctane** | | **Dextran** | | **DAO/DEX_5.0_-CNGs** | |
| --- | --- | --- | --- | --- | --- |
| **Position (cm^−1^)** | **Common** | **Position (cm^−1^)** | **Common** | **Position (cm^−1^)** | **Common** |
| 3362 | N–H stretch | 3399 | O–H stretch | 3399 | O–H stretch |
| 2924 | C–H stretch | 2916 | C–H stretch | 2930 | C–H stretch |
| 2857 | C–H stretch | 1646 | vibration of carboxyl group | 1649 | C=N stretching |
| 1583 | N–H bend | 1340 | O–H bend | 1565 | C=C stretching |
| 1494 | C–H bend | 1270 | O–H bend | 1340 | O–H bend |
| 1292 | C–N stretch | 1111 | C–O and C–C vibration at the C-4 position of glucose residue | 1270 | O–H bend |
|  |  | 1157 | C–O–C; glycosidic bonds | 1157 | C–O–C; glycosidic bonds |
|  |  | 1008 | α-1,6-glycosidic bond | 1111 | C–O and C–C vibration at the C-4 position of glucose residue |
|  |  | 912 | α-glycosidic bond | 1008 | α-1,6-glycosidic bond |

**Tab. S3** The hydrodynamic diameters and zeta potentials of DAO/DEX_0.5_-CNGs, DAO/DEX_1.5_-CNGs, DAO/DEX_5.0_-CNGs, and Spd-CQDs after being incubated in 5 mM sodium phosphate buffer (pH 7.4) or phosphate-buffered saline (PBS, pH 7.4) for 1 h.

| **DAO:DEX** | **Sodium Phosphate** | |  | | | **PBS** | |
| --- | --- | --- | --- | --- | --- | --- | --- |
|  | **Hydrodynamic Diameter (nm)** | **Zeta Potential (mV)*^a^*** | |  | **Hydrodynamic Diameter (nm)*^a^*** | | **Zeta Potential (mV)*^a^*** |
| DAO/DEX_0.5_-CNGs | 206.4 ± 7.8 | 0.8 ± 0.2 | |  | 192.7 ± 6.5 | | 0.1 ± 1.2 |
| DAO/DEX_1.5_-CNGs | 321.0 ± 45.9 | 0.6 ± 0.5 | |  | 316.5 ± 47.4 | | −0.2 ± 0.8 |
| DAO/DEX_5.0_-CNGs | 568.3 ± 22.1 | 0.6 ± 0.6 | |  | 580.2 ± 85.8 | | −1.1 ± 0.42 |
| Spd-CQDs | 38.8 ± 1.0 | 42.7 ± 3.2 | |  | 202.2 ± 19.1 | | 15.9 ± 0.9 |

**Tab. S4** Primer sequences used for RT-PCR of *Litopenaeus vannamei* immune-related genes.

| **Gene** | **Primer name** | **Sequence 5’→3’** | **Genbank** | **General Function** |
| --- | --- | --- | --- | --- |
| *LGBP* | LGBP qFw | CGGCAACCAGTACGGAGGAAC | EU102286 | The pathogen-associated molecular patterns, which recognize lipopolysaccharide and β-1,3-glucan, and subsequently trigger immunity. |
|  | LGBP qRv | GTGGAAATCATCGGCGAAGGAG |  |  |
| *ALF* | ALF qFw | CTGTGGAGGAACGAGGAGAC | DQ208705 | The polypeptide with broad-spectrum antimicrobial activity. |
|  | ALF qRw | CCACCGCTTAGCATCTTGTT |  |  |
| *cytMnSOD* | SOD qFw | ATCCACCACACAAAGCATCA | DQ029053 | The producer of superoxide radicals. |
|  | SOD qRv | AGCTCTCGTCAATGGCTTGT |  |  |
| *LYZ* | LYZ qFw | GAAGCGACTACGGCAAGAAC | AF425673 | An antimicrobial enzyme that disrupts the integrity of bacterial cell walls. |
|  | LYZ qRv | AACCGTGAGACCAGCACTCT |  |  |
| *EF1α* | EF1α qFw | GCCAGGTATGGTTGTCAACTTTG | GU136229 | Eukaryotic translation elongation factor 1 alpha; housekeeping gene. |
|  | EF1α qRv | GCCACGCTTCAGATCCTTCA |  |  |

**Additional References**

1. Jian H-J, Wu R-S, Lin T-Y, Li Y-J, Lin H-J, Harroun SG, et al. Super-cationic carbon quantum dots synthesized from spermidine as an eye drop formulation for topical treatment of bacterial keratitis. ACS Nano. 2017;11:6703−16.
2. Magoč T, Salzberg SL, FLASH: fast length adjustment of short reads to improve genome assemblies. Bioinformatics. 2011;27:2957−63.
3. Caporaso JG, Kuczynski J, Stombaugh J, Bittinger K, Bushman FD, Costello EK, et al. QIIME allows analysis of high-throughput community sequencing data. Nat Methods. 2010;7:335−6.
4. Bokulich NA, Subramanian S, Faith JJ, Gevers D, Gordon JI, Knight R, et al. Quality-filtering vastly improves diversity estimates from illumina amplicon sequencing. Nat Methods. 2013;10:57−9.
5. Haas BJ, Gevers D, Earl AM, Feldgarden M, Ward DV, Giannoukos G, et al. Chimeric 16S rRNA sequence formation and detection in Sanger and 454-pyrosequenced PCR amplicons. Genome Res. 2011;21:494−504.
6. Edgar RC, Haas BJ, Clemente JC, Quince C, Knight R, UCHIME improves sensitivity and speed of chimera detection. Bioinformatics. 2011;27:2194−200.
7. Edgar RC. Search and clustering orders of magnitude faster than BLAST. Bioinformatics. 2010;26:2460−61.
8. Edgar RC. UPARSE: highly accurate OTU sequences from microbial amplicon reads. Nat Methods. 2013;10:996−8.
9. Quast C, Pruesse E, Yilmaz P, Gerken J, Schweer T, Yarza P, et al. The SILVA ribosomal RNA gene database project: improved data processing and web-based tools. Nucleic Acids Res. 2013;41:D590−6.
10. Yilmaz P, Parfrey LW, Yarza P, Gerken J, Pruesse E, Quast C, et al. The SILVA and “all-species living tree project (LTP)” taxonomic frameworks. Nucleic Acids Res. 2014;42:D643−8.
